# Clinician-Guided Deep Learning Segmentation of Skull Base Pneumatization on Computed Tomography Using 3D Slicer and MONAI Label

**DOI:** 10.3390/life16081284

**Published:** 2026-08-03

**Authors:** Cristian-Norbert Ionescu, Gergő Ráduly, Marian Pop, Karin Ursula Horváth, Dan Iovănescu, Gheorghe Mühlfay

**Affiliations:** 1 Department of Neurosurgery, County Emergency Clinical Hospital of Târgu Mureș, 50 Gheorghe Marinescu Street, 540136 Târgu Mureș, Romania; 2 Doctoral School, George Emil Palade University of Medicine, Pharmacy, Science and Technology of Târgu Mureș, 38 Gheorghe Marinescu Street, 540139 Târgu Mureș, Romania; 3 Department of Anatomy, George Emil Palade University of Medicine, Pharmacy, Science and Technology of Târgu Mureș, 38 Gheorghe Marinescu Street, 540139 Târgu Mureș, Romania; 4 Department of Radiology, County Emergency Clinical Hospital of Târgu Mureș, 50 Gheorghe Marinescu Street, 540136 Târgu Mureș, Romania; 5 Department of Radiology ME2, George Emil Palade University of Medicine, Pharmacy, Science and Technology of Târgu Mureș, 38 Gheorghe Marinescu Street, 540139 Târgu Mureș, Romania; 6 Department of Ophthalmology, Mureș County Clinical Hospital, 6 Bernády György Street, 540072 Târgu Mureș, Romania; 7 Department of Ophthalmology, George Emil Palade University of Medicine, Pharmacy, Science and Technology of Târgu Mureș, 38 Gheorghe Marinescu Street, 540139 Târgu Mureș, Romania; 8 Department of ENT, Pius Brînzeu County Emergency Clinical Hospital of Timișoara, 156 Liviu Rebreanu Boulevard, 300723 Timișoara, Romania; 9 Department of ENT George Emil Palade University of Medicine, Pharmacy, Science and Technology of Târgu Mureș, 38 Gheorghe Marinescu Street, 540139 Târgu Mureș, Romania

**Keywords:** artificial intelligence, MONAI, MONAI Label, mastoid pneumatization, segmentation, radiomics, cranial base pneumatization, skull base surgery, convolutional neural network segmentation

## Abstract

Skull base pneumatization is anatomically variable and clinically relevant to temporal bone and transsphenoidal surgical corridors, but manual volumetric segmentation is time-consuming. This retrospective pilot study evaluated a clinician-guided deep learning workflow for mastoid and sphenoid sinus compartment segmentation on bone computed tomography. Images were curated and annotated in 3D Slicer using MONAI Label and separate three-dimensional SegResNet models. The mastoid development dataset comprised 122 side-cases, with 28 reserved side-cases; the sphenoid dataset comprised 117 development and 17 reserved examinations. The best internal-validation Dice scores were 0.8660 for the mastoid model and 0.8750 for the sphenoid model. In AI-assisted correction cohorts, mean Dice ranged from 0.9539 to 0.9691 for mastoid and from 0.9347 to 0.9426 for sphenoid. In independently annotated subsets, AI-to-expert Dice was 0.8083–0.8097 for mastoid and 0.8339–0.8473 for sphenoid, while interobserver Dice was 0.8003 and 0.9136, respectively. AI assistance reduced mean segmentation time by 86.5% for mastoid and 81.4% for sphenoid. These findings support clinician-supervised AI segmentation as an efficient starting point for volumetric assessment.

## 1. Introduction

The temporal bone is one of the most complex regions of the human skull base because of its anatomical variability and the density of functional structures within and around it, including auditory, vestibular, neural, vascular, and muscular elements. Ontogenically, it is classically formed by squamous, tympanic, petrous, mastoid, and accessory components [1]. Because of this complexity, the temporal bone and its surrounding bony and functional structures represent both a diagnostic challenge and a surgical corridor for otologic, neurotologic, and combined skull base approaches to the middle and posterior cranial fossae [2].

Understanding skull base anatomy is essential for avoiding functional morbidity during surgery. Narrow operative corridors and high-stakes decisions mean that millimetric errors may cause severe deficits. Dynamic changes in bony structures can alter conventional anatomy and the relationships of critical axial structures [3]. Cranial-base pneumatization is one such variation. Pneumatization of the temporal bone and sphenoid sinus may alter relationships between air compartments and adjacent neurovascular structures, thereby influencing surgical exposure, routes of disease spread, imaging interpretation, and the safety margins available during microsurgical or endoscopic approaches [4].

The concept of hyperpneumatization of the paranasal sinuses and cranial base has appeared in the medical literature since the beginning of the twentieth century and remains relevant to otorhinolaryngology; maxillofacial surgery; radiology; and, more recently, neurosurgery [5]. Cranial base pneumatization may be influenced by local ventilation, developmental factors, inflammation, and pressure-related mechanisms [3,4,6,7], including possible effects of atmospheric conditions. The mastoid air-cell system is routinely assessed on computed tomography (CT), but usually qualitatively. Volumetric analysis could support quantitative phenotyping, the investigation of disease associations, and surgical planning [5,8,9,10,11,12].

The sphenoid sinus, located centrally at the skull base, provides an important surgical corridor for minimally invasive neurosurgical and multidisciplinary skull base procedures. It is adjacent to critical structures, including the internal carotid arteries, cavernous sinuses, optic nerves, pituitary fossa, and other neural structures [13]. In rare selected cases, markedly enlarged air spaces may be associated with neurological or neuro-ophthalmological manifestations [14,15,16,17,18]. Sphenoid sinus hyperpneumatization, internal septations, and lateral recess development may influence the safety of transsphenoidal or transclival approaches [13,19]. In particular, an intrasphenoidal septum may attach to a thinned lateral wall or carotid prominence, creating potential risk during septal removal, drilling, or rostrum manipulation [13].

High-resolution computed tomography (HRCT) provides excellent bone–air contrast and remains the standard imaging modality for evaluating sphenoid sinus and mastoid anatomy. Manual delineation of these spaces is labor-intensive and susceptible to observer variability. Mastoid air cells are irregular, trabeculated, and spatially variable; they may extend toward the petrous or zygomatic regions and may contain sclerotic or fluid-density components. The sphenoid sinus is centrally located and morphologically heterogeneous and may contain variable septations, mucosal thickening, fluid-density changes, or pathological tissue. These characteristics make both structures clinically important but technically challenging segmentation targets [8,11,20,21,22].

Recent advances in artificial intelligence (AI) and medical image segmentation provide opportunities to convert skull base CT interpretation into quantitative analysis. Quantitative assessment of aerated skull base compartments may support volumetry, anatomical risk mapping, disease monitoring, and future investigations of subtle density or mucosal changes. However, many AI tools remain difficult for clinicians to adapt and integrate because they require programming expertise, dedicated graphics processing unit (GPU) infrastructure, or fixed single-task plugins that cannot be tailored to the patient-specific anatomical question [20,21].

Open-source platforms such as 3D Slicer and the Medical Open Network for Artificial Intelligence (MONAI) may offer a practical alternative by supporting clinician-guided active learning. In this workflow, an automatic prediction is generated, reviewed, corrected by an expert physician, and incorporated into subsequent model development. This approach is suitable for skull base anatomy, where expert anatomical and surgical judgment remains essential and segmentation targets may be too variable for purely automated development [23,24,25].

MONAI Label, SegResNet, and clinician-in-the-loop segmentation are established technologies. Accordingly, this study does not propose a novel neural-network architecture or segmentation algorithm. Its contribution lies in applying and evaluating a unified clinician-guided workflow for two morphologically distinct cranial base pneumatization targets: the mastoid air-cell system and the sphenoid sinus. The study further combines automatic segmentation, expert correction, independent manual reference masks, interobserver assessment, annotation-time analysis, and exploratory volumetric and CT attenuation measurements within the same workflow.

This pilot study aimed primarily to develop and evaluate a clinician-guided segmentation workflow for temporal bone pneumatization on high-resolution CT using MONAI Label [23]. The secondary aim was to assess whether the same workflow could be extended to sphenoid sinus segmentation on full-head bone CT. The clinical question was whether an established deep learning framework, combined with expert supervision, could delineate these anatomically irregular air spaces reproducibly while reducing the time required for fully manual segmentation. The primary hypothesis was that unchanged model-generated masks would demonstrate substantial spatial and volumetric agreement with independently created expert manual masks and that AI-assisted correction would significantly reduce annotation time. The secondary hypothesis was that independently created and AI-assisted expert masks would demonstrate high interobserver agreement and support reproducible volumetric and CT attenuation analyses. The study therefore evaluates a clinician-in-the-loop application of an established architecture rather than proposing a novel deep learning method.

## 2. Materials and Methods

### 2.1. Study Design

This ongoing pilot research was designed as a retrospective model development and validation study of clinician-guided deep learning segmentation of skull base pneumatization on computed tomography. The primary arm evaluated segmentation of the temporal bone air-cell system and its extensions. The secondary arm assessed whether the same assisted workflow could be adapted to sphenoid sinus segmentation on full-head bone CT examinations.

### 2.2. Data Collection and Imaging Material

**Figure 1:**
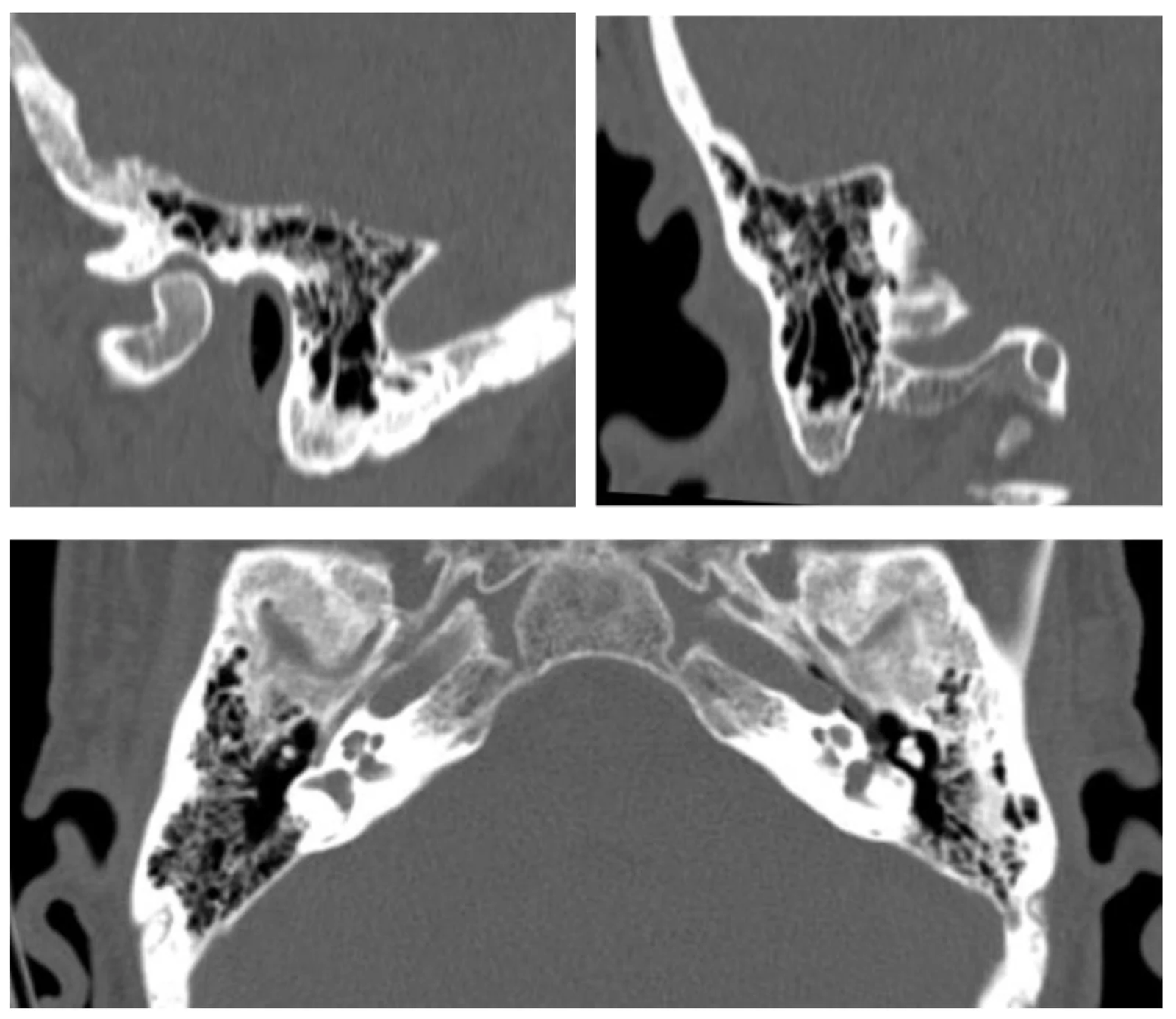
Mastoid and temporal bone CT dataset in sagittal, coronal, and axial planes.

**Figure 2:**
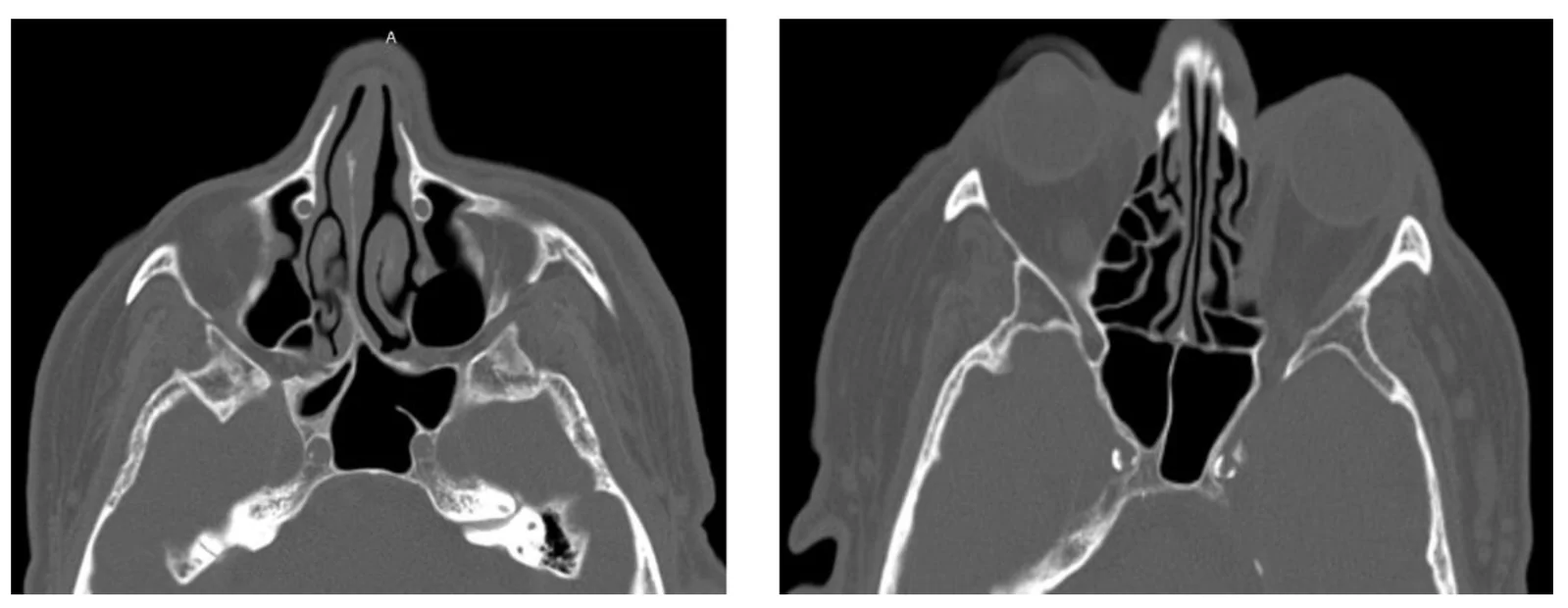
Sphenoid sinus bone CT dataset in axial planes.

In this retrospective pilot study, head CT examinations were selected separately for the two project arms. The investigators selected non-consecutive examinations following eligibility screening, without computer-generated randomization. All examinations were performed in the Department of Radiology at the County Emergency Clinical Hospital of Târgu Mureș (SCJU Târgu Mureș) using three CT scanners with adaptive acquisition settings based on individual patient characteristics. We selected 75 head CT examinations with bone reconstructions for the mastoid project (Figure 1) and 134 full-head CT examinations with bone reconstructions for the sphenoid project (Figure 2), yielding 209 source examinations in total.

Source data were acquired using Siemens Healthineers SOMATOM X.cite CT system (Siemens Healthcare GmbH, Erlangen, Germany), GE Healthcare Revolution Frontier, and GE Healthcare Optima CT660 scanners (GE Medical Systems, operating as GE HealthCare, Waukesha, WI, USA). Verified Digital Imaging and Communications in Medicine (DICOM) metadata showed predominantly bone-kernel reconstructions, including GE BONEPLUS and Siemens Hr36s kernels. Median tube voltage was 120 kVp for the Revolution Frontier, 120 kVp for the Optima CT660, and 100 kVp for the SOMATOM X.cite. Median reconstructed slice thickness ranged from 0.625 to 1.5 mm according to scanner and source series, with a median in-plane pixel spacing of approximately 0.49–0.51 mm.

CT series were included when they provided a bone-window or bone-kernel reconstruction; sufficient target anatomy coverage; adequate spatial resolution for skull base segmentation; interpretable anatomy; and no severe motion, duplicated-volume artifacts, or corrupted geometry. Traumatic and postoperative examinations were excluded to avoid confounding the assessment of normal anatomical variability.

For mastoid model development, preference was given to thin-slice, high-resolution CT examinations with adequate bone–air contrast and complete visualization of the temporal bone.

For sphenoid segmentation, full-head bone CT examinations were included when the sphenoid region and surrounding skull base were adequately visualized. Cases with difficult but clinically meaningful anatomy, including fluid-filled or inflammatory-appearing sphenoid sinuses, were retained within the development cohort when their osseous boundaries remained interpretable.

### 2.3. Ethical Approval

This retrospective study was conducted in accordance with the Declaration of Helsinki and was approved by the Institutional Ethics Committee of the County Emergency Clinical Hospital of Târgu Mureș (approval no. 17233, 27 August 2024; approval no. 13572, 29 June 2026). The requirement for individual informed consent was waived because of the retrospective study design, the use of anonymized imaging data, and the absence of additional patient intervention.

### 2.4. Image Curation and Dicom Conversion

Clinical DICOM series were screened to identify technically suitable bone CT reconstructions. Selection considered the reconstruction description, anatomical region, acquisition type, reconstruction kernel, slice thickness, image count, and anatomical coverage. Scouts, topograms, localizers, maximum-intensity projections, secondary multiplanar reconstructions, non-cranial examinations, vascular or venous reconstructions, and non-bone series were excluded.

Eligible DICOM series were converted to Neuroimaging Informatics Technology Initiative (NIfTI) format using SimpleITK version 2.5.3 (Insight Software Consortium; https://simpleitk.org/; accessed on 31 July 2026). Image spacing, physical origin, direction, and orientation metadata were retained during conversion. Post-conversion quality control included verification of dimensions, voxel spacing, field of view, anatomical orientation, z-axis ordering, and anatomical plausibility. Different preprocessing strategies were used for the two targets: automatic side-specific temporal bone cropping for mastoid segmentation and full-head CT preservation for sphenoid segmentation. Detailed image geometry is reported in Supplementary Table S1.

#### 2.4.1. Mastoid Side-Crop Preparation and Canonicalization

The mastoid workflow used side-specific temporal bone crops to reduce non-target anatomy and increase the relative representation of the segmentation target. CT-series eligibility and final image quality were reviewed by the clinical investigators; however, crop localization was automatic and rule-based. Crop coordinates were not manually defined and were not derived from annotation or ground-truth boundaries.

Each CT volume was first reoriented to the left-posterior-superior (LPS) coordinate system. An image-based foreground mask was generated using a lower attenuation threshold of −500 HU. Connected-component analysis was performed, and the largest component was used to estimate the head boundaries. A three-dimensional bounding box was calculated around this component and expanded by six voxels within the limits of the source image. The transverse midpoint of the detected head bounding box was used to generate symmetric left- and right-sided crops. A base lateral extent equal to approximately 55% of the detected head width was used for each side. Each crop was additionally extended by approximately 8% of the head width around the midpoint, producing a shared central overlap zone of approximately 16%. The complete anteroposterior and craniocaudal extent of the detected head bounding box was retained. Crops were generated using the SimpleITK RegionOfInterest operation. Voxel spacing and image direction were retained, while the physical origin was updated according to crop position. Left-sided crops were transformed to a right-sided canonical configuration by flipping the first and third image-index axes and subsequently reorienting the result to LPS. Right-sided crops were not mirrored and were standardized directly to LPS. The same operations were applied to corresponding images and labels. These index-space operations did not require intensity interpolation. Image-label dimensions, spacing, origin, direction, and anatomical alignment were checked after transformation. The preprocessing manifest recorded the source bounding box, crop coordinates, crop dimensions, voxel spacing, anatomical side, and canonicalization transform. Because localization depended only on CT intensity and geometry, the same procedure was applied to reserved evaluation images without access to ground-truth labels. The cropping stage therefore represented automatic rule-based preprocessing, not manual, semi-automatic, or ground-truth-derived localization.

The mastoid target was represented by one foreground class designated mastoid. Separate left and right output classes were unnecessary because all side-crops had been standardized to a common right-sided orientation.

**Figure 3:**
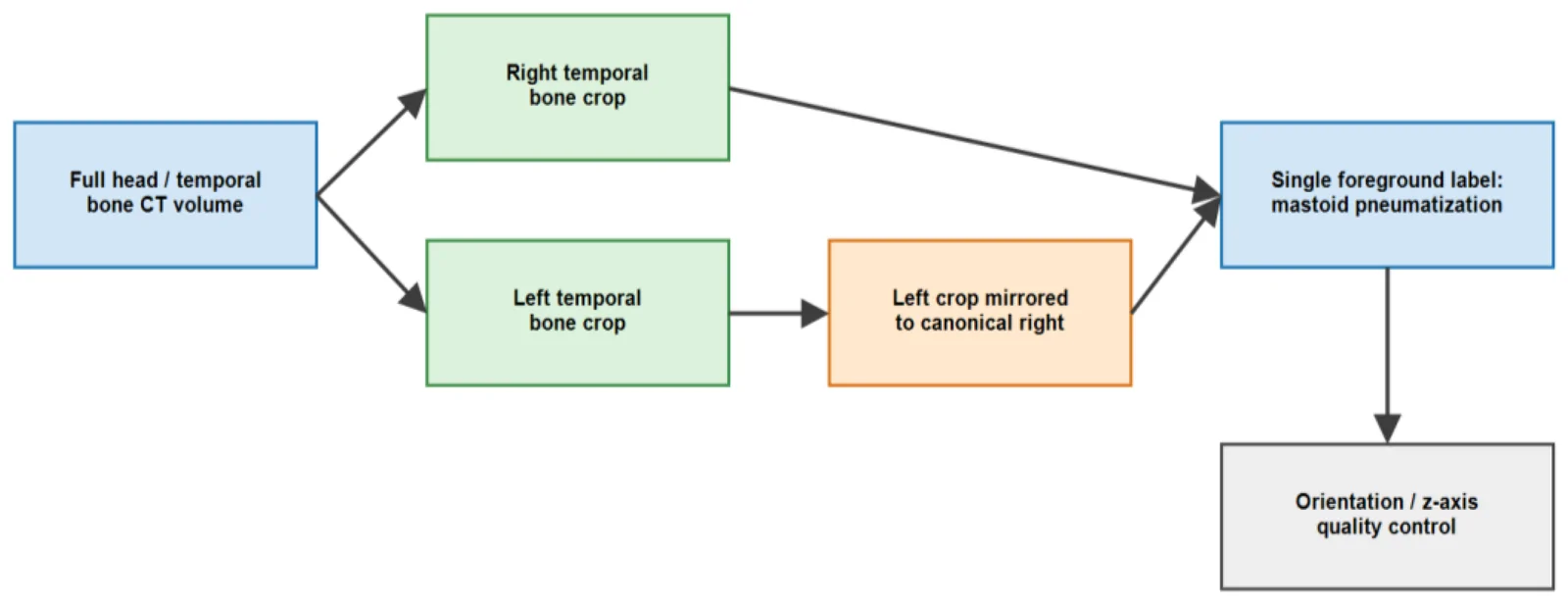
Mastoid side crop and canonicalization workflow.

The mastoid side-crop preparation and canonicalization workflow is illustrated in Figure 3.

#### 2.4.2. Sphenoid Full-Head CT Preparation

The sphenoid workflow retained complete full-head bone CT volumes because the sphenoid sinus is a central skull base structure that may extend asymmetrically across the midline. Full anatomical coverage also provided context for distinguishing the target from the ethmoid, frontal, and maxillary sinuses. No target-derived crop or manual localization was used.

The 117 frozen development volumes retained a 512 × 512 axial matrix with variable z-axis coverage. Across the final development manifest, the z-axis range was 66–375 slices, and the median matrix was 512 × 512 × 260 voxels. Median voxel spacing was approximately 0.488 × 0.488 × 0.625 mm. Variation in image extent and spacing was handled through model-level resampling and sliding-window processing. The sphenoid target was represented by one foreground class designated sphenoid.

### 2.5. Anatomical Segmentation Targets

The study targets were defined as anatomical pneumatization compartments rather than threshold collections of air-density voxels. Consequently, segmented volumes could contain air, mucosal or fluid-density content, and internal bony septations. This distinction is important because the reported volumes represent the delineated anatomical compartment and not only the aerated fraction.

For the mastoid arm, the intended target comprised the mastoid and temporal bone pneumatization compartment, including continuous extensions within the petrous, squamous, zygomatic, or posterior temporal bone regions when these belonged to the same pneumatization system. Internal osseous septa were included within the compartment mask. Opacified or sclerotic mastoid cells were also included when their location and boundaries identified them as part of the target compartment. The tympanic cavity, external auditory canal, surrounding outer cortical bone, and unrelated extracranial air were excluded.

For the sphenoid arm, the target comprised the complete bilateral sphenoid sinus compartment, including communicating recesses and internal content within its outer osseous boundaries. Internal sphenoid septa were included within the compartment mask, together with air, mucosal, or fluid-density content. The outer sphenoid walls, ethmoid, frontal, and maxillary sinuses; nasopharyngeal air space; cavernous sinus; and other adjacent non-target structures were excluded.

### 2.6. Monai Label and Neural Network Architecture

**Figure 4:**
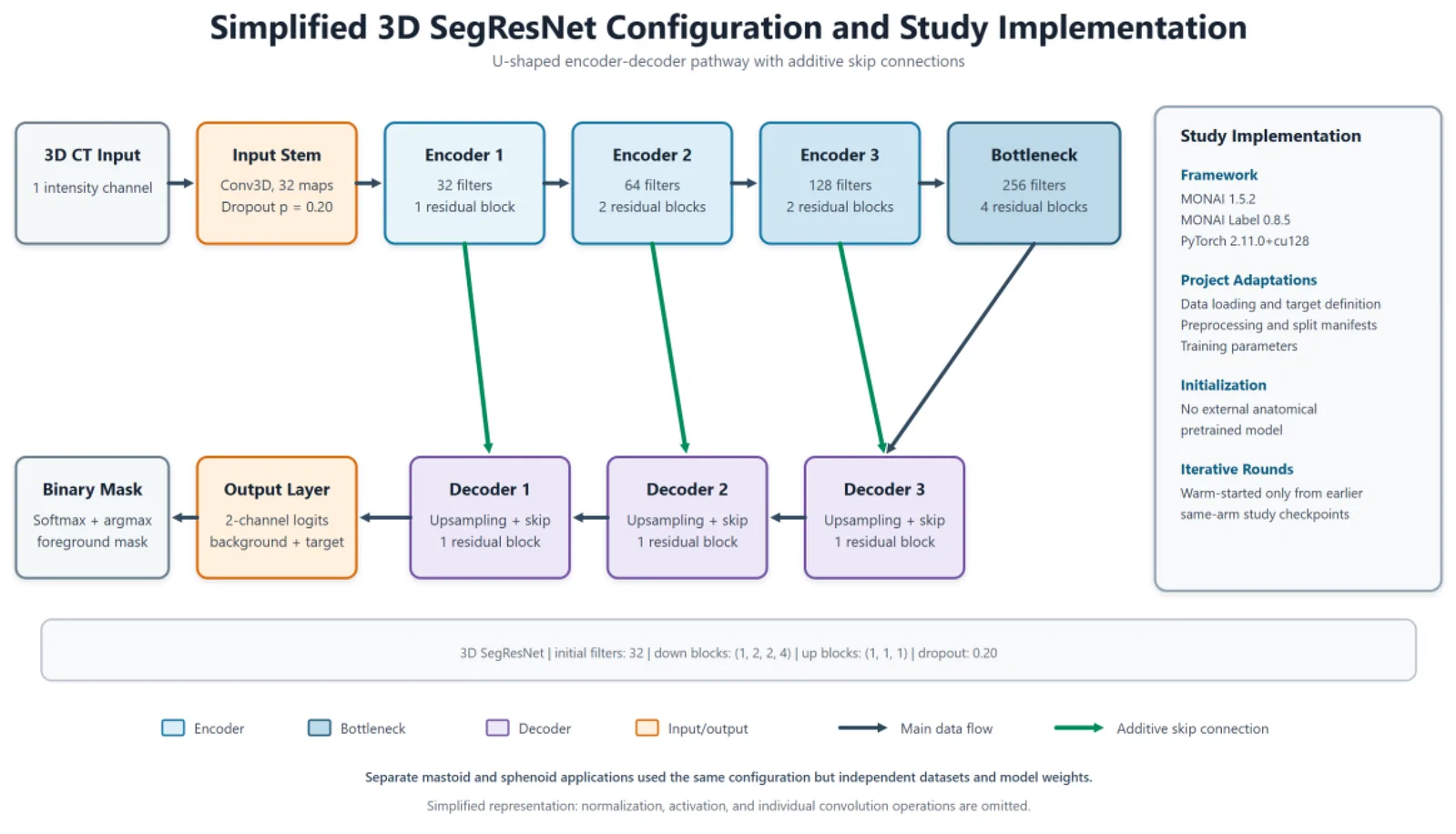
Simplified three-dimensional SegResNet configuration and study implementation. A single-channel CT input underwent an initial three-dimensional convolution followed by dropout. Four residual encoder levels, displayed as three encoder stages and one bottleneck, used block depths of (1, 2, 2, 4) and filter widths of 32, 64, 128, and 256, respectively. Three decoder stages, with block depths of (1, 1, 1), were connected to the corresponding encoder levels through additive skip connections. Dark blue arrows indicate the main data-flow pathway, while green arrows indicate additive skip connections. Light blue boxes represent encoder stages, darker blue represents the bottleneck, purple represents decoder stages, orange represents input/output processing, and gray represents the CT input and final binary mask. The output layer generated background and target logits, followed by softmax/argmax post-processing to obtain the binary mask. The MONAI Label 0.8.5 framework was adapted through project-specific data loading, target definition, preprocessing, split manifests, and training parameters. No external anatomical pretrained model was used; iterative rounds were initialized only from earlier checkpoints within the same anatomical study arm. The mastoid and sphenoid applications used the same configuration but independent datasets and model weights. Normalization, activation, and individual convolution operations are omitted for clarity.

Separate MONAI Label applications and model lineages were maintained for the mastoid and sphenoid targets. The segmentation network was a three-dimensional SegResNet residual encoder–decoder convolutional neural network implemented with MONAI version 1.5.2 (Project MONAI; https://monai.io/; accessed on 31 July 2026) and PyTorch version 2.11.0+cu128 (PyTorch Foundation; https://pytorch.org/; accessed on 31 July 2026). The network received one CT intensity channel and generated two output logits corresponding to background and foreground. The study configuration used 32 initial filters, residual downsampling blocks of (1, 2, 2, 4), upsampling blocks of (1, 1, 1), and a dropout probability of 0.2 (Figure 4) [23,24,26]. A simplified summary of the three-dimensional SegResNet architecture is provided in Figure S2.

The official MONAI Label 0.8.5 framework was adapted through project-specific data loading, target definitions, preprocessing, split manifests, and training parameters. No externally pretrained anatomical model was used. Later iterative rounds could be initialized from earlier checkpoints generated within the same study arm.

#### 2.6.1. Annotation

Manual annotation, model-output review, and expert correction were performed in 3D Slicer 5.10.0 using the MONAI Label 0.8.5 extension. Images were assessed in axial, coronal, and sagittal planes. Initial masks were created manually; subsequent development cases were segmented using an iterative clinician-in-the-loop process in which model predictions were reviewed and corrected before submission. New blocks of corrected labels were incorporated into later model-development rounds.

**Figure 5:**
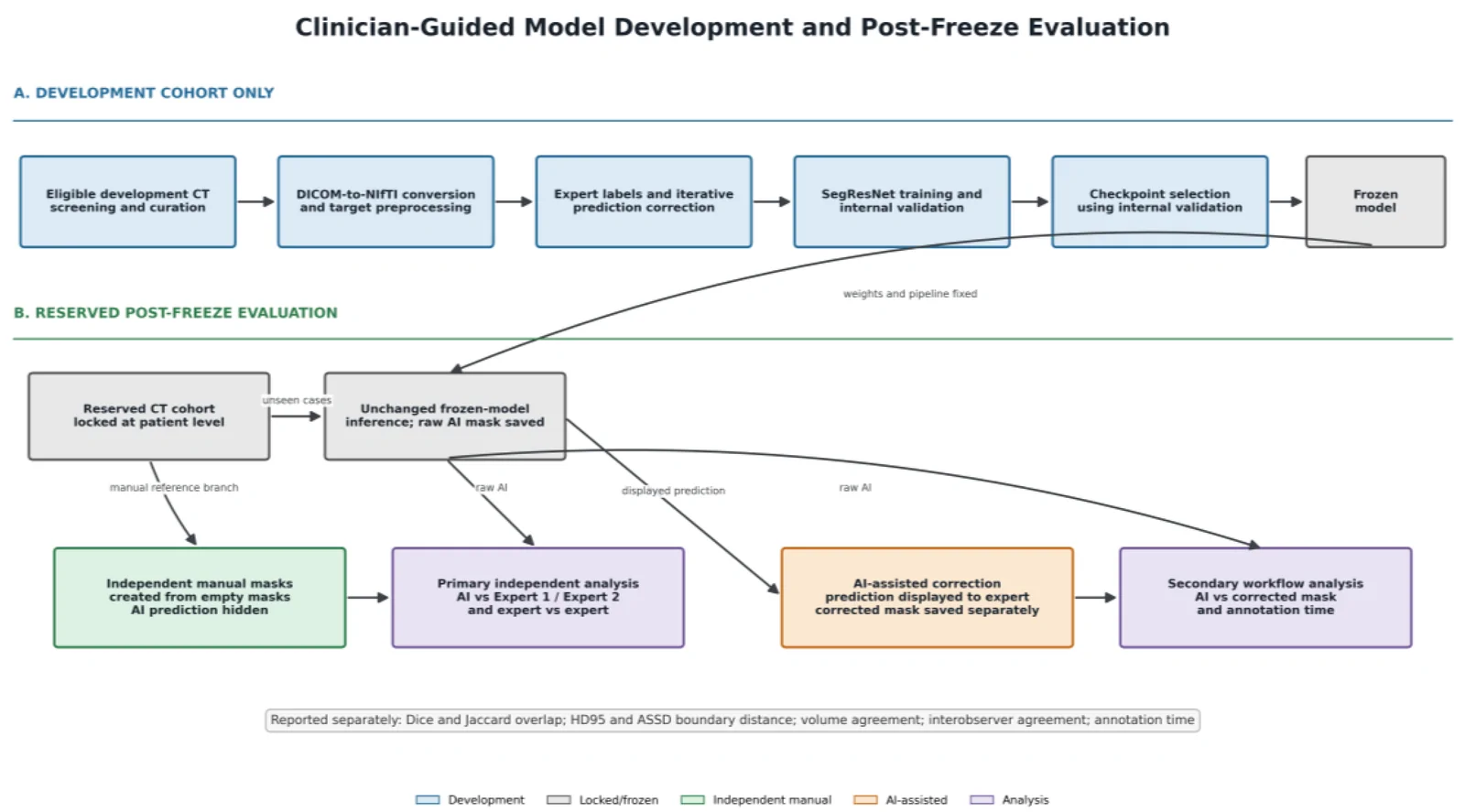
Clinician-guided model-development and post-freeze evaluation workflow. The development cohort was used for image curation, preprocessing, expert annotation, iterative prediction correction, SegResNet training, internal validation, and checkpoint selection. After model freezing, the reserved patient-level cohort underwent unchanged model inference. Independent reference masks were created without displaying the AI prediction and were used for the primary AI-to-expert and interobserver analyses. AI-assisted correction was evaluated separately as a secondary workflow analysis using saved raw predictions and expert-corrected masks. Dice, Jaccard index, 95th-percentile Hausdorff distance (HD95), average symmetric surface distance (ASSD), volume agreement, interobserver agreement, and annotation time were reported separately.

This iterative correction process was used to clarify the operational region of interest and improve consistency across variable anatomy. It is reported as a practical annotation and model development workflow, not as evidence of a novel network architecture (Figure 5) [25]. A compact overview of the clinician-guided segmentation pipeline is provided in Figure S1.

Additional workflow details and the complete source code are provided in the Supplementary Materials.

#### 2.6.2. Training Configuration

Training used a clinician-guided interactive three-dimensional (3D) workflow. Initially, 15 cases in each cohort were segmented manually and submitted for training. After the first predictive models were generated in MONAI Label 0.8.5, subsequent development cases were reviewed and corrected in batches of 10–20, followed by retraining after each submitted label block. This process allowed refinement of the operational region of interest (ROI) across variable anatomical patterns [23,24]. Iterative prediction correction and retraining involved only development cases and excluded the reserved evaluation cohorts.

The training and reporting structure was informed by previous CT-based studies of sphenoid sinus geometry and pneumatization classification [20,21] and by established reporting principles for medical-image segmentation. The present study reports cohort size, expert annotation, network architecture, dataset separation, epoch number, validation interval, and performance using overlap, surface-distance, and volumetric agreement metrics. Its methodological contribution is the clinician-guided volumetric segmentation of two skull base pneumatization compartments using MONAI Label and 3D Slicer rather than a new neural-network architecture.

Training was performed separately for the mastoid and sphenoid projects to avoid mixing anatomical targets, image formats, and preprocessing strategies. Both models used AdamW optimization with a weight decay of 1 × 10^−5^ and Dice–cross-entropy loss. The learning rate was 1 × 10^−5^ for the mastoid model and 5 × 10^−6^ for the sphenoid model. CT intensities were scaled from −1000 to 2000 HU. Training used a target spacing of 0.5 × 0.5 × 0.625 mm, 64 × 64 × 64-voxel patches, a batch size of one, and full-volume sliding-window inference for validation. Validation was performed every five epochs. The patch size and batch size were selected to maintain stable execution within the available GPU memory.

Internal-validation Dice represented model-development performance and was used exclusively for checkpoint selection. The 122 mastoid development side-cases comprised 95 training and 27 internal-validation cases. Because max_val_records was set to 20, each internal-validation cycle evaluated the first 20 records from the fixed 27-case list. This computational limit did not affect final expert validation, for which all 28 reserved mastoid side-cases were analyzed. The sphenoid development split comprised 102 training and 15 internal-validation examinations.

Both models were developed and evaluated using the same local GPU-based environment. Detailed hardware, software, CUDA, training-time, and annotation-time specifications are provided in the Supplementary Materials. Model inference time was not prospectively recorded and was therefore not reported.

#### 2.6.3. Training Metrics

The Dice similarity coefficient was defined as Dice = 2TP/(2TP + FP + FN), where TP represented true-positive voxels, FP represented false-positive voxels, and FN represented false-negative voxels. The Jaccard index was defined as TP/(TP + FP + FN), sensitivity as TP/(TP + FN), and precision as TP/(TP + FP). Relative volume error was defined as 100 × (V_auto − V_reference)/V_reference, where V_auto was the automatic segmentation volume and V_reference was the relevant independent manual or AI-assisted corrected reference volume for the explicitly identified analysis. Manual and assisted reference categories were analyzed separately and were not pooled.

Boundary agreement was evaluated using the 95th-percentile Hausdorff distance (HD95, mm) and average symmetric surface distance (ASSD, mm). HD95 was selected instead of maximum Hausdorff distance because it is less sensitive to isolated outlier voxels, while ASSD quantified the average bidirectional boundary discrepancy between the evaluated masks.

The complete metric set included the Dice similarity coefficient, Jaccard index, sensitivity/recall, precision/positive predictive value, HD95 in millimeters, ASSD in millimeters, surface Dice at a 1 mm tolerance, automatic segmentation volume, reference-mask volume, relative and absolute relative volume error, false-positive volume, and false-negative volume [27].

##### Segmentation Metrics and Analysis

Spatial overlap was quantified using the Dice similarity coefficient and Jaccard index (intersection over union). Sensitivity (recall) and precision (positive predictive value) were calculated voxel-wise. Boundary agreement was assessed using the 95th-percentile Hausdorff distance (HD95), average symmetric surface distance (ASSD), and surface Dice at a tolerance of 1 mm. All distance metrics were calculated in physical units after geometry-safe alignment of the evaluated masks.

Absolute volumetric agreement between experts was assessed using ICC(2,1), a two-way random-effects intraclass correlation coefficient for absolute agreement based on single measurements. Bland–Altman analysis was used to estimate the mean volume difference and 95% limits of agreement. The 95% confidence intervals for mean Dice were estimated using 100,000 bootstrap resamples; intervals for the bilateral mastoid assisted workflow were clustered at the patient level.

Segmentation time was measured from successful case loading to completed mask submission. Fully manual and AI-assisted correction times were retained separately. Matched manual and AI-assisted correction times were compared using the Wilcoxon signed-rank test; unmatched timing summaries were considered exploratory. Cases affected by server failure, interrupted loading, or an absent automatic prediction were excluded from timing analysis.

##### Exploratory Volumetric and Densitometric Analysis

Submitted compartment masks were used for exploratory volumetric and CT attenuation analysis. Non-overlapping descriptive intervals were defined as air density below −300 HU, mucosa-like density from −300 to less than −50 HU, fluid-like density from −50 to +100 HU, intermediate density above +100 to +150 HU, and dense or bony-septation density above +150 HU. These thresholds were used only for descriptive mask characterization and were not interpreted as diagnostic classifications of inflammation, infection, fungal disease, or tumor.

#### 2.6.4. Reserved Evaluation Cohorts and Test Isolation

After applying the imaging eligibility criteria, the clinical investigators selected evaluation cases from the eligible pool without reference to model predictions or segmentation performance. The selection was non-consecutive, and no computer-generated randomization sequence was used.

For the mastoid arm, 14 patients were assigned to the reserved evaluation cohort before bilateral side processing. Both temporal bones from each source examination remained in the same cohort, producing 28 evaluation side-cases. None of these 28 images or labels was included in the 122-side development dataset, model training, or internal validation. No patient in the reserved mastoid cohort contributed an opposite-side crop or another examination to the development cohort.

For the sphenoid arm, 17 full-head bone CT examinations were assigned to the reserved evaluation cohort. None of these images or labels were included in the 117-case development dataset, model training, or internal validation. Training and internal validation were mutually exclusive, and the reserved evaluation cohort remained independent of both subsets.

Model and checkpoint selection was based exclusively on the corresponding internal validation subsets. Images, labels, metrics, and failure cases from the reserved mastoid and sphenoid cohorts did not influence gradient updates, hyperparameter selection, preprocessing, post-processing, or checkpoint selection. The frozen models and unchanged inference pipelines were applied to the reserved cohorts only for final expert evaluation.

#### 2.6.5. Manual Reference and AI-Assisted Evaluation

The original post-training workflow compared unchanged predictions with expert-corrected masks. Because correction of a displayed prediction can introduce anchoring and incorporation bias, these data were reclassified as AI-assisted workflow analyses.

During revision, two clinical experts independently delineated matched cases from empty masks while blinded to the automatic predictions and to each other’s segmentations. Expert 1 was the first author, a PGY-5 neurosurgery resident, and Expert 2 was the sixth author, a senior otorhinolaryngologist. This AI-blinded analysis included eight mastoid side-cases and ten sphenoid full-head CT volumes from the reserved evaluation cohorts. Manual interobserver agreement was calculated by comparing the Expert 1 and Expert 2 masks. The eight mastoid and ten sphenoid examinations were selected by the clinical investigators from the reserved cohorts before examination of case-level AI performance metrics; selection was non-consecutive, and no computer-generated randomization sequence was used.

The frozen models were applied unchanged to the same manually annotated cases. Model performance was calculated separately as AI versus Expert 1 and AI versus Expert 2. AI-assisted, expert-corrected masks were analyzed separately from fully manual references. For the mastoid arm, corrected masks from both experts were available for all 28 reserved side-cases, permitting assisted-correction interobserver analysis. Fully manual masks, unchanged AI outputs, and assisted corrected masks were stored as distinct data categories and were not pooled.

## 3. Results

### 3.1. Dataset Composition

The mastoid development dataset comprised 122 side-specific temporal bone cases derived from 61 CT examinations after the exclusion of three inadequate right-sided cases. A separate reserved cohort comprised 28 sides from 14 additional CT examinations. Splitting was performed at the patient level: both temporal bones from the same examination were assigned to the same cohort, and no patient contributed one side to development and the opposite side to evaluation.

The sphenoid development dataset comprised 117 full-head bone CT examinations. Seventeen additional examinations were reserved for AI-assisted workflow evaluation. Reserved mastoid and sphenoid cases were investigator-selected from examinations meeting the predefined eligibility criteria, without formal algorithmic randomization, and were not used to update model weights. The independent manual subsets were evaluated only after model freezing. Detailed CT slice burden and segmented-slice extent are reported in the Supplementary Materials.

### 3.2. Mastoid Segmentation and Training Results

The complete mastoid development history contained 43 recorded runs and 1595 cumulative epochs. This total describes the iterative development workload rather than the exposure of each case to one uninterrupted 1595-epoch training process. It included exploratory single-side, left-only, right-only, combined-side, repaired, rollback, interrupted, and final refinement runs. Only completed or checkpoint-preserved runs with auditable split and metric records informed the final configuration; interrupted or configuration-test runs were not presented as final performance experiments. The complete mastoid training history, including exploratory, recovery, and final runs, is summarized in Table S2.

During early mastoid development, the project produced a reproducible sequence of informative single-side models. Validation Dice increased from 0.8613 in a completed 40-epoch run to 0.8840 in a second 40-epoch run and 0.9244 in a completed 40-epoch run. A subsequent run reached a preserved peak of 0.9291 but stopped after 30 of 40 planned epochs; the next complete 40-epoch run reached 0.9285. A later 50-epoch run declined to 0.8829, preceding the more marked deterioration that subsequently prompted a separate label- and geometry-integrity audit.

**Figure 6:**
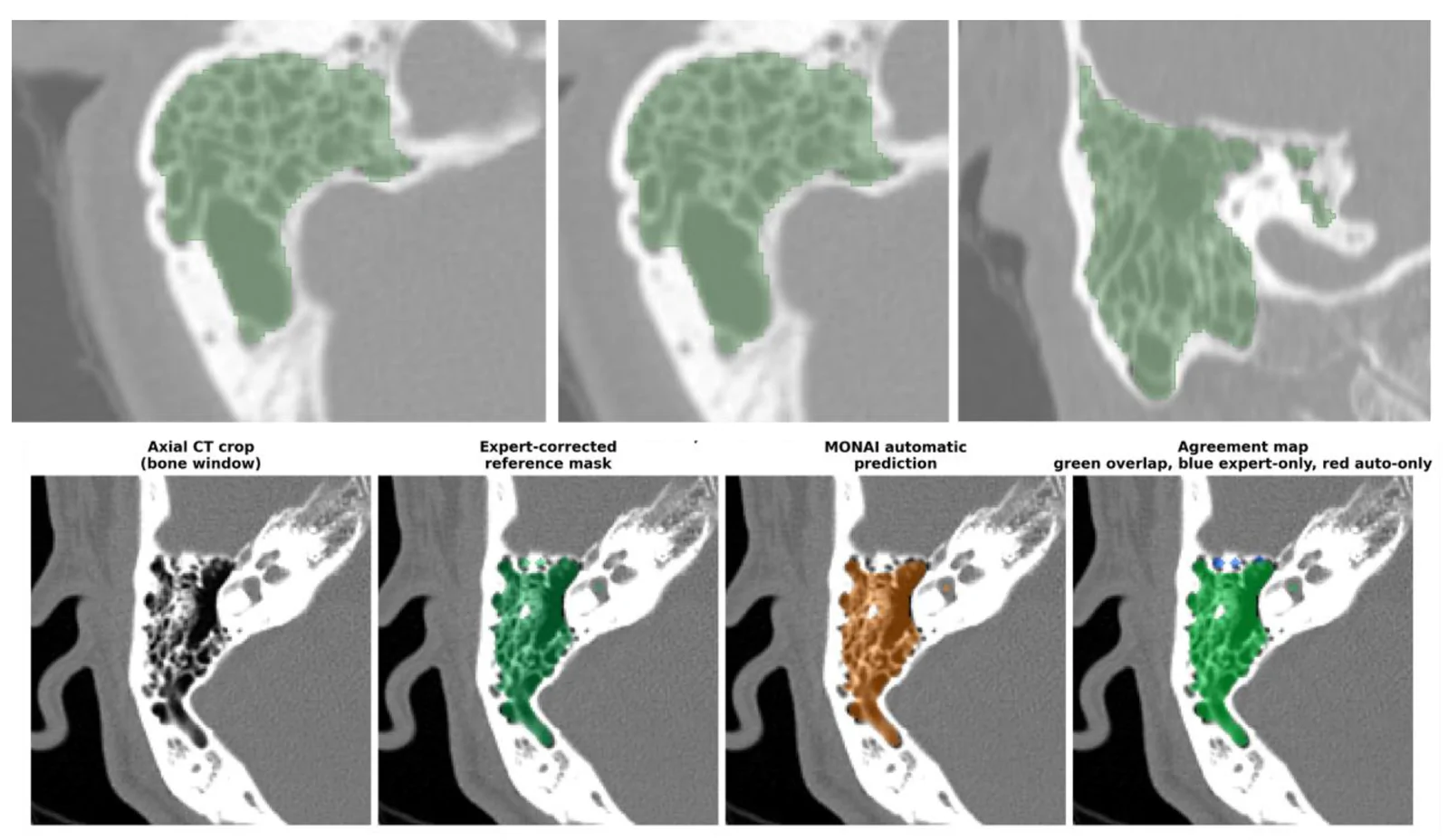
In-training 0.92 internal validation Dice mask used mid-project. Representative mastoid segmentation on bone-window CT. The upper row shows the semi-transparent expert-corrected mask in three orthogonal anatomical planes. The lower row presents, from left to right, the original axial temporal-bone crop, the expert-corrected reference mask in green, the MONAI automatic prediction in orange, and the voxel-wise agreement map. In the agreement map, green represents overlap between the expert and automatic masks (true-positive voxels), blue represents expert-only regions indicating AI under-segmentation (false-negative voxels), and red represents automatic-only regions indicating AI over-segmentation (false-positive voxels).

These six key early-development milestones represented 240 reached epochs and established that the anatomical target was learnable. The historical lineage with validation Dice of approximately 0.92 was used operationally for the right-sided arm: its predictions and expert-corrected outputs provided the high-quality right-sided mask basis used during dataset consolidation and subsequent combined-side development (Figure 6).

Thus, these early runs were not merely discarded experiments; they materially contributed to the labels used for the right-sided component of the final dataset. They are included in the 1595-epoch historical development total. However, they are not all included in the 120-epoch directly inherited checkpoint lineage because label provenance and neural-network weight ancestry are distinct, and not every early checkpoint directly initialized the final combined model.

After the side-handling and label-integrity audit, the mastoid workflow was rebuilt in stages rather than restarted as a single long run. A right-side recovery run reached a best validation Dice of 0.6448. Revalidated left-side training then improved from 0.4885 to 0.7644 and 0.8437. The integration of the curated left-side labels with the right-side label basis derived from the historical workflow with validation Dice of approximately 0.92 produced a combined best validation Dice of 0.8644, followed by the final stable value of 0.8660. This staged recovery supported preservation of the useful right-side work while correcting the affected left-side and geometry pipeline.

The final mastoid configuration retained the three-dimensional SegResNet architecture and AdamW optimization used during development. Images were resampled to 0.5 × 0.5 × 0.625 mm; training used 64 × 64 × 64-voxel regions, four sampled regions per image, a 3:1 positive-to-negative sampling ratio, and a batch size of 1. Inference used a 96 × 96 × 96-voxel sliding window with 0.1 overlap. These settings balanced target coverage and local GPU memory limits while preserving the thin trabecular boundaries of the mastoid compartment.

**Figure 7:**
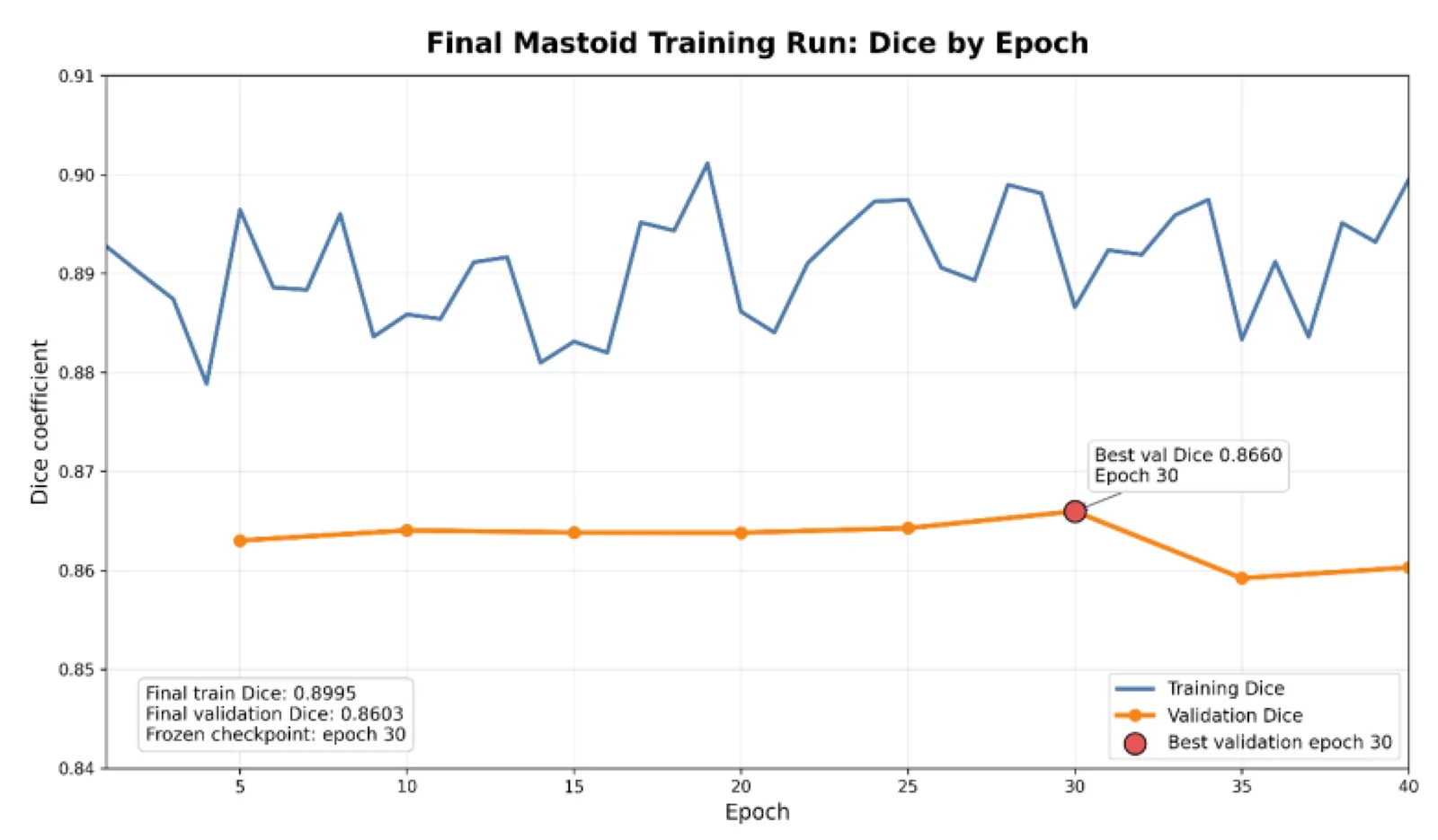
Final selected mastoid training run. The blue line shows training Dice by epoch, the orange line shows internal-validation Dice assessed every five epochs, and the red marker identifies the checkpoint with the best internal-validation Dice retained for evaluation.

The final result-bearing mastoid run used 95 side-cases for optimization and 27 side-cases allocated to internal validation. Because the MONAI Label configuration used max_val_records = 20, a maximum of 20 validation records contributed to each validation cycle; this explains the difference between the 27 allocated cases and 20 effective records. The run completed all 40 planned epochs in 3 h 53 min 35 s and was validated every five epochs. The best internal-validation Dice was 0.8660 at epoch 30; the training and validation Dice scores at epoch 40 were 0.8995 and 0.8603, respectively. The epoch-30 checkpoint, rather than the last epoch, was frozen for evaluation (Figure 7).

The final locked mastoid dataset contained a total of 6630 segmented axial slices across the 122 side-cases. The median number of segmented slices per side-case was 56.5, with a mean of 54.3 and a range of 13 to 89 slices. The median segmented z-axis span was 38.1 mm, with a mean of 36.3 mm.

#### 3.2.1. Mastoid Expert-Comparison Results

The frozen mastoid model, selected at an internal-validation Dice of 0.8660, showed high agreement with the Expert 1 corrected reference masks. Across the 28 reserved side-cases, the mean and median Dice similarity coefficients for this comparison were 0.9691 and 0.9794, respectively. The mean Jaccard index was 0.9419. Boundary agreement was also favorable, with a median HD95 of 0.9468 mm and a mean surface distance of 0.3099 mm. The mean relative volume error was −5.7866%, indicating a mild tendency toward under-segmentation relative to the expert-corrected final labels.

In the second-expert AI-assisted evaluation, the original 28-case analysis reported model agreement with the Expert 1 corrected masks. Using the same preserved model outputs, Expert 2 independently corrected the displayed predictions. The mean raw-AI-versus-Expert 2 corrected-mask Dice was 0.9539 (median, 0.9824; bootstrap 95% CI, 0.9044–0.9830). Agreement between the Expert 1- and Expert 2-assisted final masks was 0.9468 (median, 0.9745; bootstrap 95% CI, 0.9028–0.9730).

In the blinded manual mastoid evaluation, eight reserved mastoid side-cases were segmented independently by both experts without displaying the AI prediction. The mean interobserver Dice was 0.8003 (95% CI, 0.7790–0.8225), and volume agreement was ICC(2,1) = 0.9854 (95% CI, 0.8415–0.9966), with a Bland–Altman bias of −0.004 mL and 95% limits of agreement from −0.873 to 0.865 mL. The mean AI-versus-expert Dice was 0.8083 for Expert 1 (95% CI, 0.7735–0.8411) and 0.8097 for Expert 2 (95% CI, 0.7780–0.8408).

**Figure 8:**
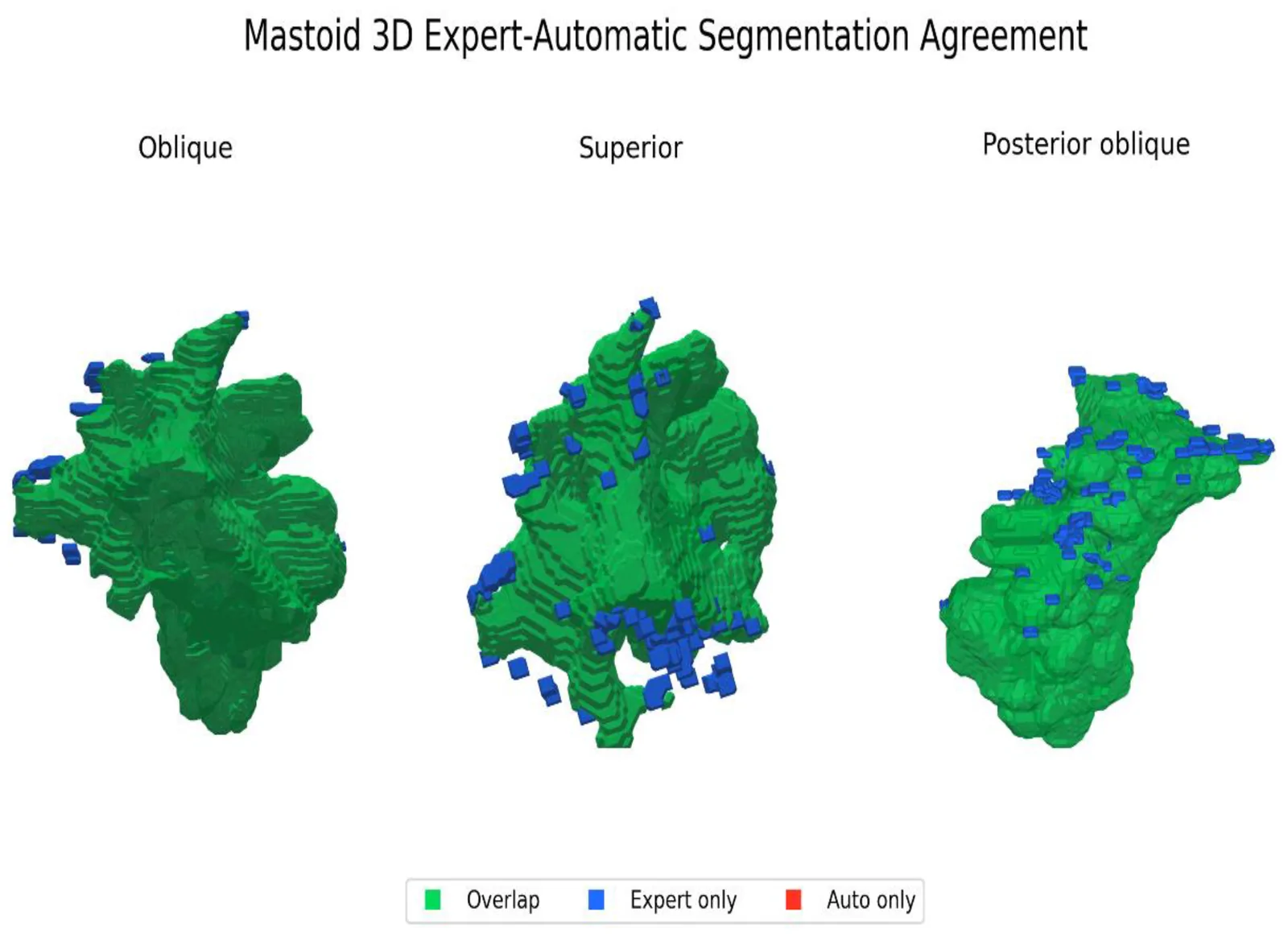
Expert vs. automatic segmentation overlapping on 3D Mastoid model in 3 views from the final validation pool (green—overlap, blue—expert only, red—auto only).

Figure 8 shows a three-dimensional representation of the mastoid compartment comparing the automatic prediction with the expert reference mask and illustrating the predominantly conservative under-segmentation pattern.

#### 3.2.2. Exploratory Temporal Bone Pneumatization Patterns

Exploratory extension-pattern screening was performed using a rule-based morphometric proxy from the locked side-crop masks. Superior temporal squama extension was defined as mask extension into the superior quartile of the side-crop, posterior extension as extension into the posterior quartile, zygomatic/anterior extension as extension into the anterior quartile, and petrous/medial extension as side-aware mask extension toward the medial crop quartile. A total of 58 extension-pattern detections were recorded across the four proxies; individual side-cases could meet more than one criterion (Table 1).

**Figure 9:**
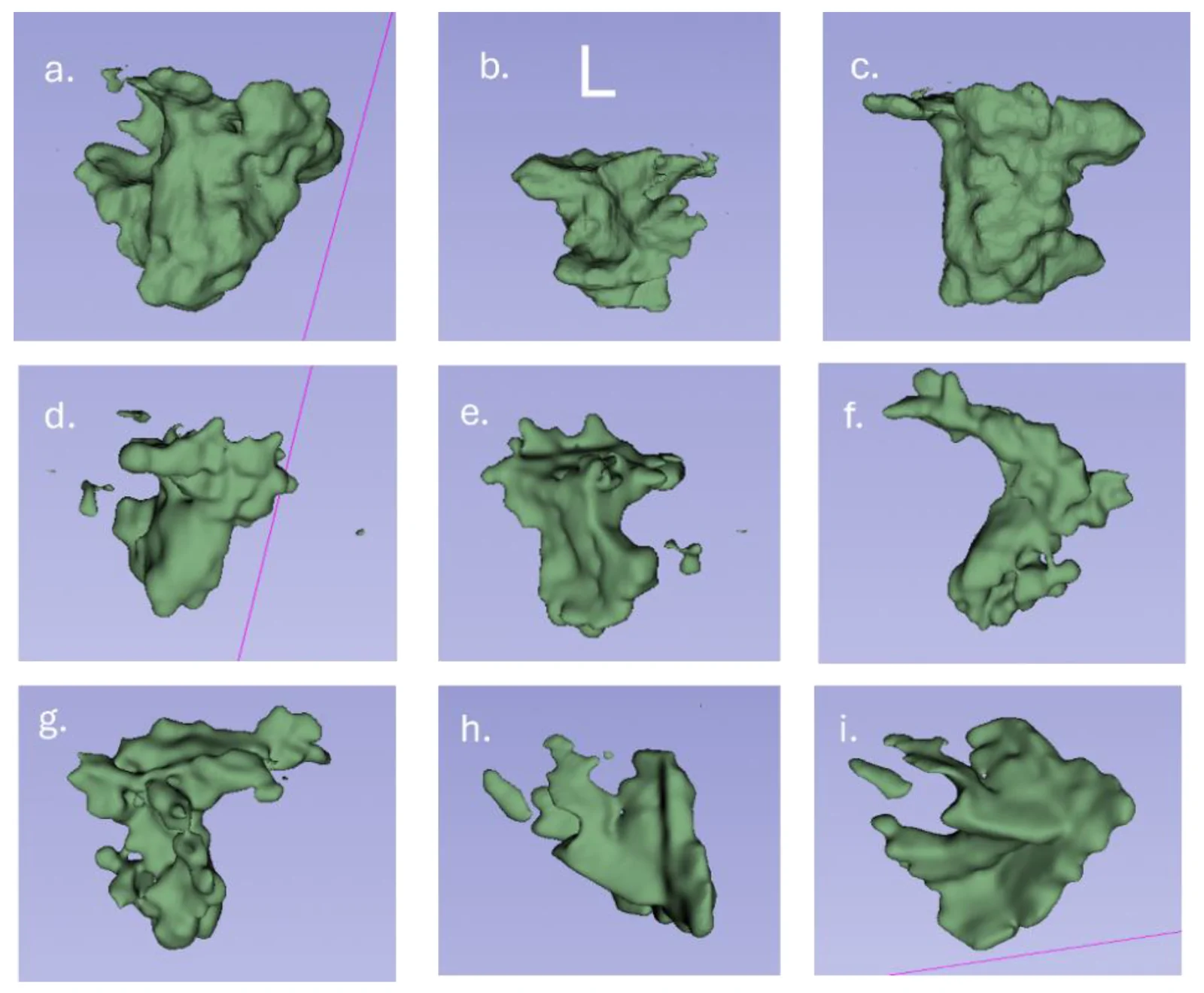
Different pneumatization patterns of the temporo-mastoid complex represented in 3D views ((**a**–**c**) lateral and oblique; (**d**–**g**) oblique; (**h**–**i**) superior views).

Representative pneumatization patterns viewed from different orientations are shown in Figure 9.

### 3.3. Sphenoid Arm Results

**Figure 10:**
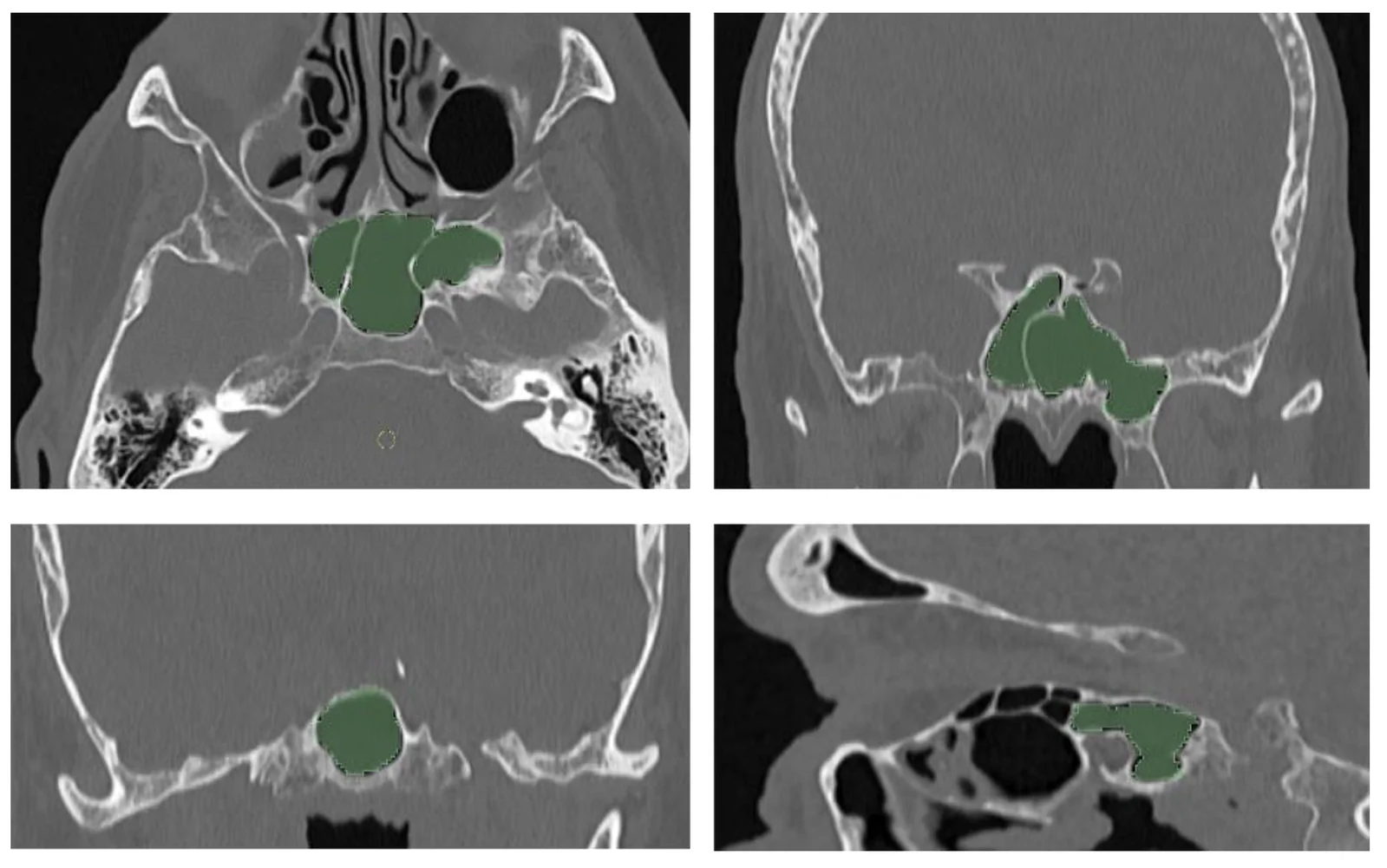
In-training automatic segmentation of the sphenoid sinus showing extensive pneumatization of lateral compartments (green label of the sinus—in training automatic segmentation).

The final sphenoid model was developed using 117 expert-submitted labels, including 102 training and 15 internal-validation cases. Across the complete development process, 332 epochs were recorded, of which 215 were used directly in the final results. The selected checkpoint achieved the best internal-validation Dice of 0.8750 at epoch 10 and was frozen for the reserved AI-assisted and blinded-manual evaluations (Figure 10). The mean and median sphenoid compartment volumes were 9.40 and 8.77 mL, respectively (range, 1.79–21.82 mL). Detailed training history, CT slice characteristics, and segmented-slice extent are provided in the Supplementary Materials. Detailed sphenoid training durations and epoch histories are provided in Table S3. De-tailed sphenoid image and submitted-label characteristics are provided in Table S4.

#### Sphenoid Expert-Validation Results

The 17 reserved cases were automatically segmented using the frozen model selected at an internal-validation Dice of 0.8750. Automatic masks were saved separately, the cases were corrected by the expert in 3D Slicer, and the submitted expert-corrected masks were compared with the automatic masks.

**Figure 11:**
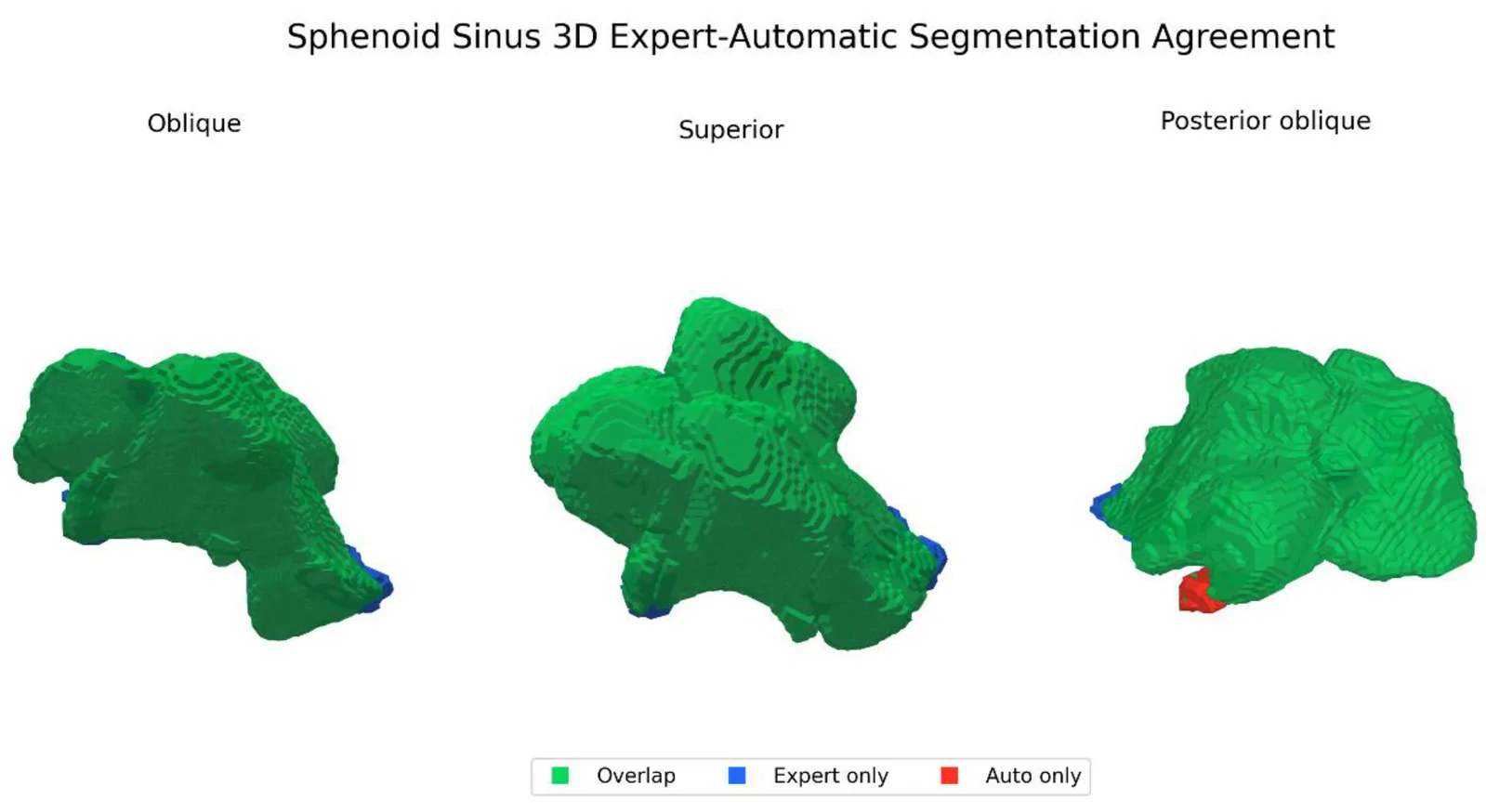
Expert vs. automatic segmentation overlapping on 3D Sphenoid model in 3 views from final validation pool (green–overlap, blue–expert only, red–auto only).

Across the 17-case sphenoid expert-validation cohort, mean and median Dice were 0.9426 and 0.9408, respectively. Mean and median Jaccard indices were 0.8937 and 0.8882. Boundary agreement was favorable, with a median HD95 of 1.875 mm and a median average symmetric surface distance (ASSD) of 0.331 mm. Median relative volume error was −11.18%, indicating predominantly conservative under-segmentation, as illustrated by the three-dimensional model in Figure 11. A representative best-performing sphenoid automatic-versus-manual overlap on native bone CT is shown in Figure S3.

In the second-expert AI-assisted evaluation, the original 17-case analysis reported model agreement with the Expert 1 corrected masks. Using the same preserved model outputs, Expert 2 independently corrected the displayed predictions. The mean raw-AI-versus-Expert 2 corrected-mask Dice was 0.9347 (median, 0.9279; bootstrap 95% CI, 0.9188–0.9506). Agreement between the Expert 1- and Expert 2-assisted final masks was 0.9682 (median, 0.9708; bootstrap 95% CI, 0.9600–0.9762).

**Figure 12:**
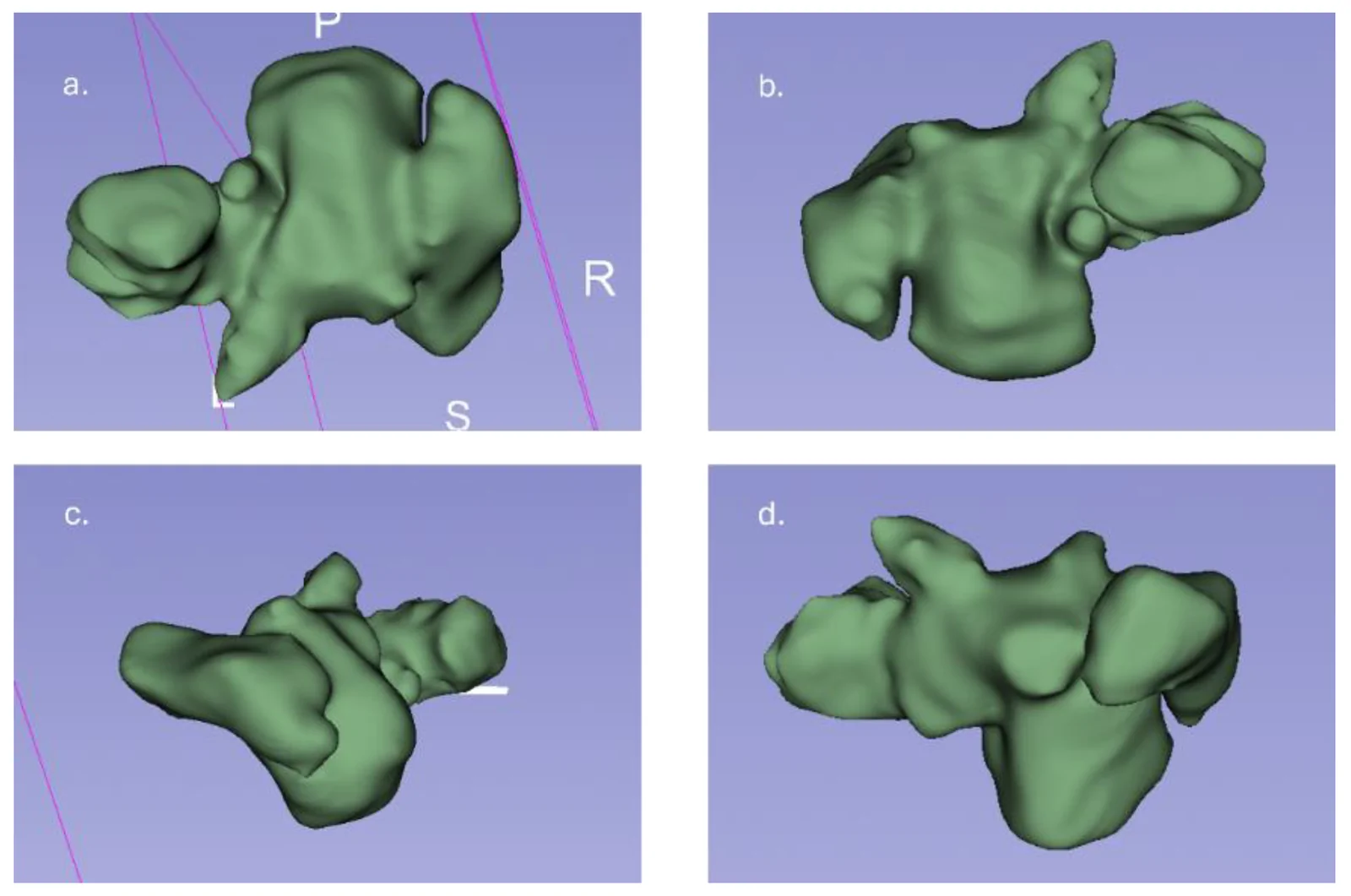
Different sphenoid sinus air compartment morphologies ((**a**). superior view, (**b**). posterior view, (**c**). oblique/lateral view, (**d**). anterior view).

In the blinded manual sphenoid evaluation, ten reserved sphenoid CT examinations were segmented independently by both experts without displaying the AI prediction. The mean interobserver Dice was 0.9136 (95% CI, 0.9047–0.9215), and volume agreement was ICC(2,1) = 0.9938 (95% CI, 0.9747–0.9970), with a Bland–Altman bias of 0.549 mL and 95% limits of agreement from −0.181 to 1.278 mL. The mean AI-versus-expert Dice was 0.8473 for Expert 1 (95% CI, 0.8041–0.8858) and 0.8339 for Expert 2 (95% CI, 0.7922–0.8718). The blinded manual analyses estimate independent model-versus-expert agreement. The higher AI-assisted values describe correction from a shared model mask and were analyzed separately. Differences in the fully manual masks occurred mainly at thin bony limits, intracavitary septa, narrow recesses, and opacified compartments. Several morphological patterns were observed in the training labels, including extensive inferolateral recess development. Representative three-dimensional renderings are shown in Figure 12.

### 3.4. Final Validation Tendencies

**Figure 13:**
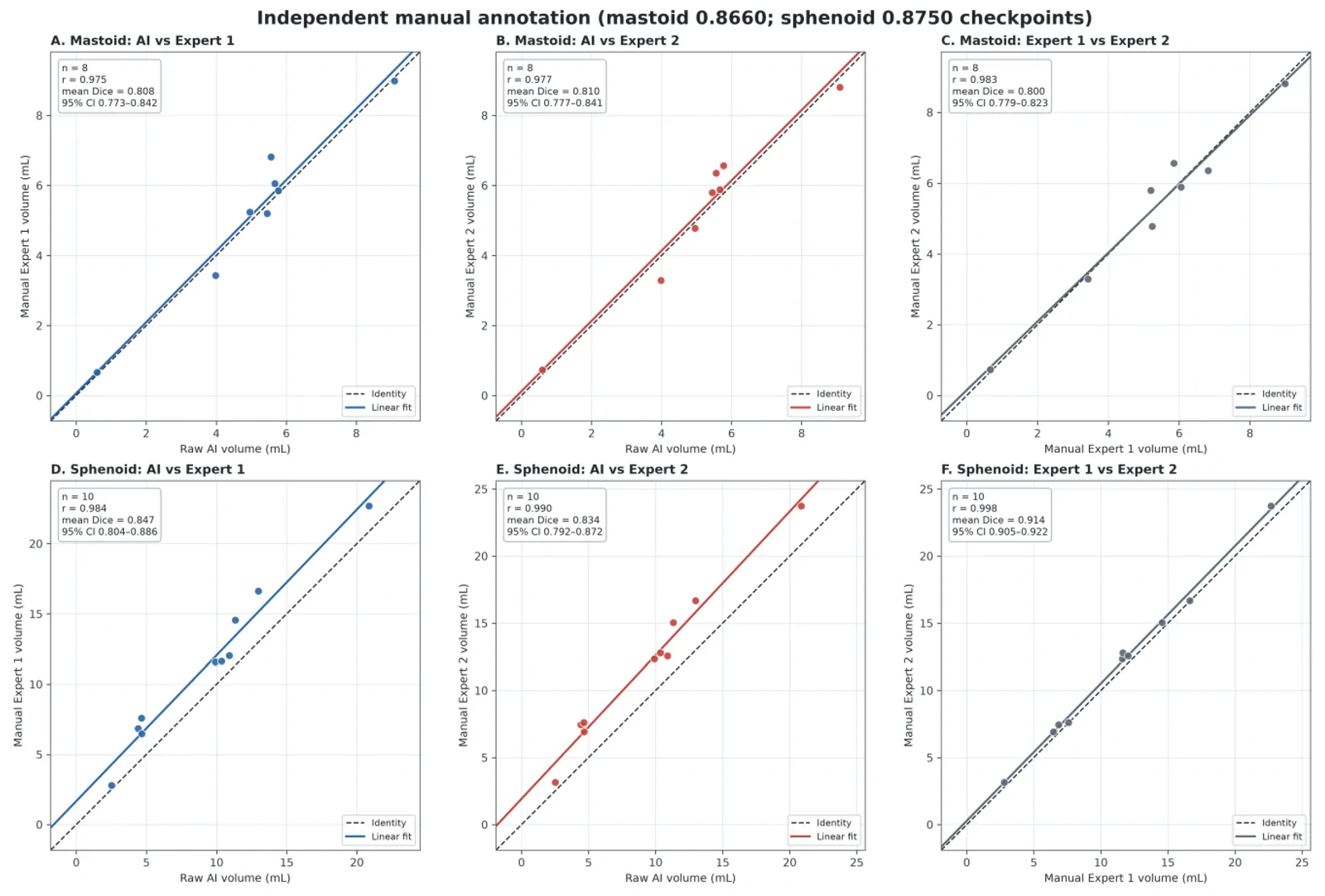
Independent manual evaluation of mastoid and sphenoid segmentation volumes. Panels (**A**–**C**) show mastoid comparisons (n = 8): AI versus Expert 1, AI versus Expert 2, and Expert 1 versus Expert 2. Panels (**D**–**F**) show the corresponding sphenoid comparisons (n = 10). Blue indicates AI–Expert 1, red indicates AI–Expert 2, and gray indicates interobserver comparisons. Each circle represents one anonymized case. The dashed line is the identity line (y = x), and the solid line is the linear regression fit. The symbol r represents Pearson’s correlation coefficient, mean Dice represents average spatial overlap, and 95% CI represents the bootstrap confidence interval for mean Dice. Sphenoid expert volumes were generally larger than AI volumes, consistent with conservative under-segmentation. AI, artificial intelligence; CI, confidence interval.

**Figure 14:**
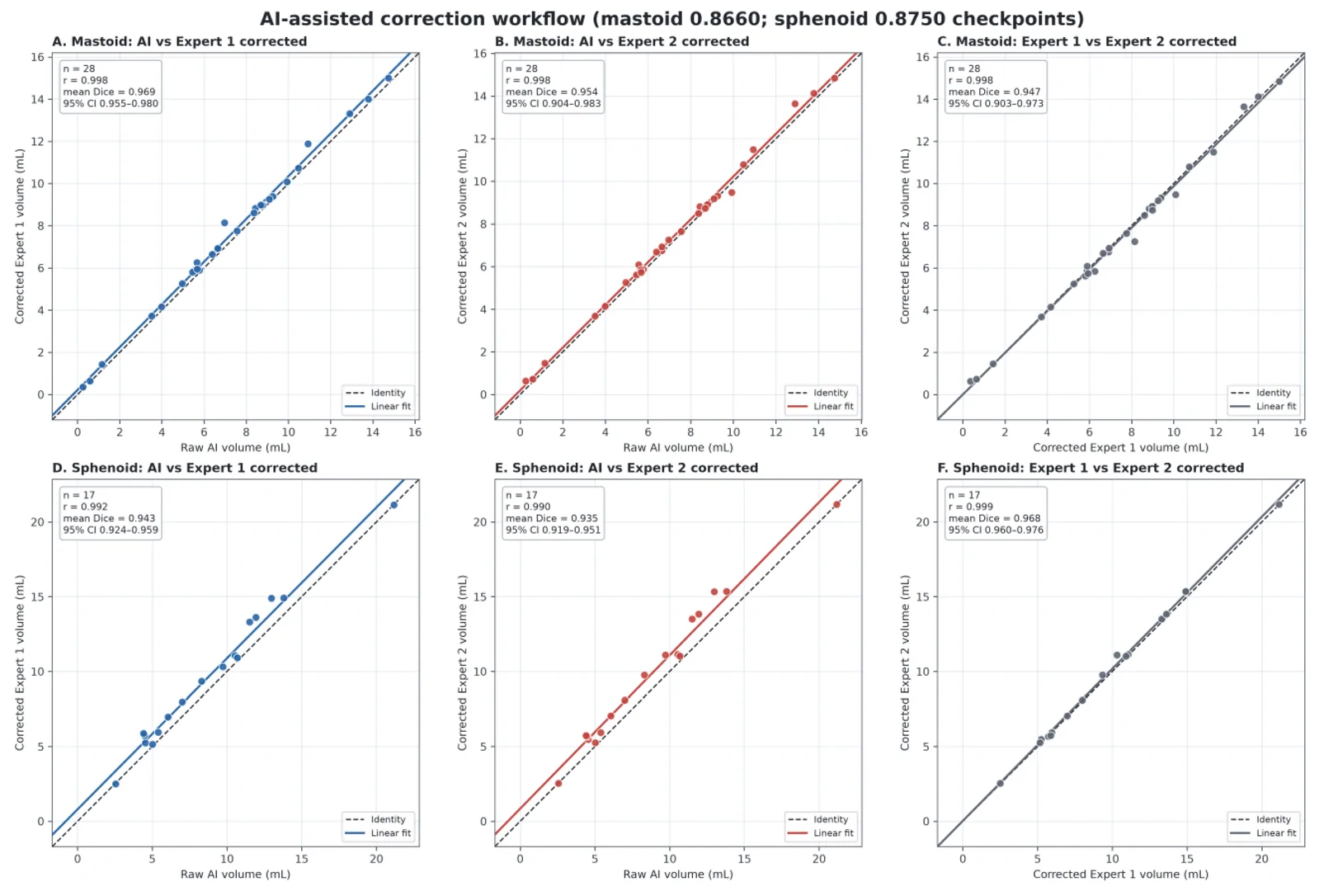
AI-assisted segmentation and expert-correction workflow. Panels (**A**–**C**) show mastoid comparisons (n = 28), and panels (**D**–**F**) show sphenoid comparisons (n = 17): raw AI versus Expert 1-corrected masks, raw AI versus Expert 2-corrected masks, and Expert 1- versus Expert 2-corrected masks. Blue, red, and gray indicate these respective comparisons. Each circle represents one anonymized case. The dashed line is the identity line, and the solid line is the linear regression fit. The symbol r denotes Pearson’s correlation coefficient, mean Dice represents spatial overlap, and 95% CI represents the bootstrap confidence interval for mean Dice. These results describe AI-assisted workflow agreement because both experts corrected a displayed model prediction.

In the blinded manual subsets, the mastoid model achieved mean Dice values of 0.8083 against Expert 1 and 0.8097 against Expert 2, comparable to the interobserver Dice of 0.8003. For the sphenoid model, mean Dice was 0.8473 against Expert 1 and 0.8339 against Expert 2, while interobserver Dice was higher at 0.9136. The sphenoid model showed high precision with lower sensitivity, indicating predominantly conservative under-segmentation. Higher Dice values were obtained in the AI-assisted cohorts: 0.9691 and 0.9539 for mastoid and 0.9426 and 0.9347 for sphenoid against the Expert 1- and Expert 2-corrected masks, respectively. However, the blinded-manual and assisted results are not directly interchangeable because they involved different cohort sizes and reference-mask construction. In the assisted workflow, both experts corrected the same displayed AI mask, providing a shared initial topology and boundary proposal. This reduced variability at thin bony limits, intracavitary septa, narrow recesses, and opacified compartments but introduced potential anchoring and incorporation bias. Therefore, the blinded manual comparisons were considered the primary independent evaluation of model performance (Figure 13), whereas the higher assisted Dice values describe the consistency and practical efficiency of the clinician-guided correction workflow (Figure 14).

#### 3.4.1. Boundary Agreement

Mastoid boundary agreement was substantially higher at the 2 mm tolerance than at 1 mm. Interobserver surface Dice increased from 0.912 at 1 mm to 0.994 at 2 mm. AI-to-expert surface Dice increased from 0.769–0.773 at 1 mm to 0.903–0.913 at 2 mm, indicating that most AI–expert boundary discrepancies were localized within approximately 2 mm. Bland–Altman analysis demonstrated negligible interobserver volume bias (−0.004 mL; 95% limits of agreement, −0.873 to 0.865 mL). AI volumes were slightly smaller than the expert volumes, with biases of 0.145 mL for Expert 1 minus AI and 0.141 mL for Expert 2 minus AI, consistent with mild conservative under-segmentation.

Sphenoid boundary agreement was also substantially higher at the 2 mm tolerance than at 1 mm. Interobserver surface Dice increased from 0.922 at 1 mm to 0.984 at 2 mm. AI-to-expert surface Dice increased from 0.714–0.757 at 1 mm to 0.894–0.903 at 2 mm, indicating that most model–expert boundary discrepancies were situated within approximately 2 mm. The Bland–Altman analysis showed an interobserver volume bias of 0.549 mL (Expert 2 minus Expert 1; 95% limits of agreement, −0.181 to 1.278 mL). Expert volumes exceeded AI volumes by a mean of 2.024 mL for Expert 1 and 2.573 mL for Expert 2, confirming a predominantly conservative AI under-segmentation pattern.

#### 3.4.2. Best- and Worst-Performing Cases

For both anatomical targets, the frozen AI models showed a predominantly conservative under-segmentation tendency compared with independently created manual masks. Agreement improved substantially when a 2 mm surface tolerance was applied, indicating that most differences were localized near compartment boundaries rather than representing major anatomical error.

For the mastoid, AI–expert volumetric bias was small, and performance was close to interobserver Dice. Disagreement mainly involved peripheral air cells, thin bony septa, and irregular extensions of the pneumatized compartment. The model generally identified the correct anatomical region but occasionally omitted small peripheral or partially opacified cells.

For the sphenoid, under-segmentation was more pronounced. Expert-defined volumes were consistently larger than AI volumes, particularly along posterior, inferior, and lateral recesses and around internal septa. Nevertheless, high surface Dice at 2 mm showed that most discrepancies remained spatially close to the manually defined boundary.

Overall, the AI models produced anatomically plausible initial masks with few extensive false-positive extensions. The principal correction required was the addition of omitted peripheral regions, supporting their use as conservative starting masks in a clinician-guided workflow rather than as fully autonomous segmentations.

Best and worst cases were selected using the mean AI-versus-manual Dice across both experts shown in Table 2.

**Figure 15:**
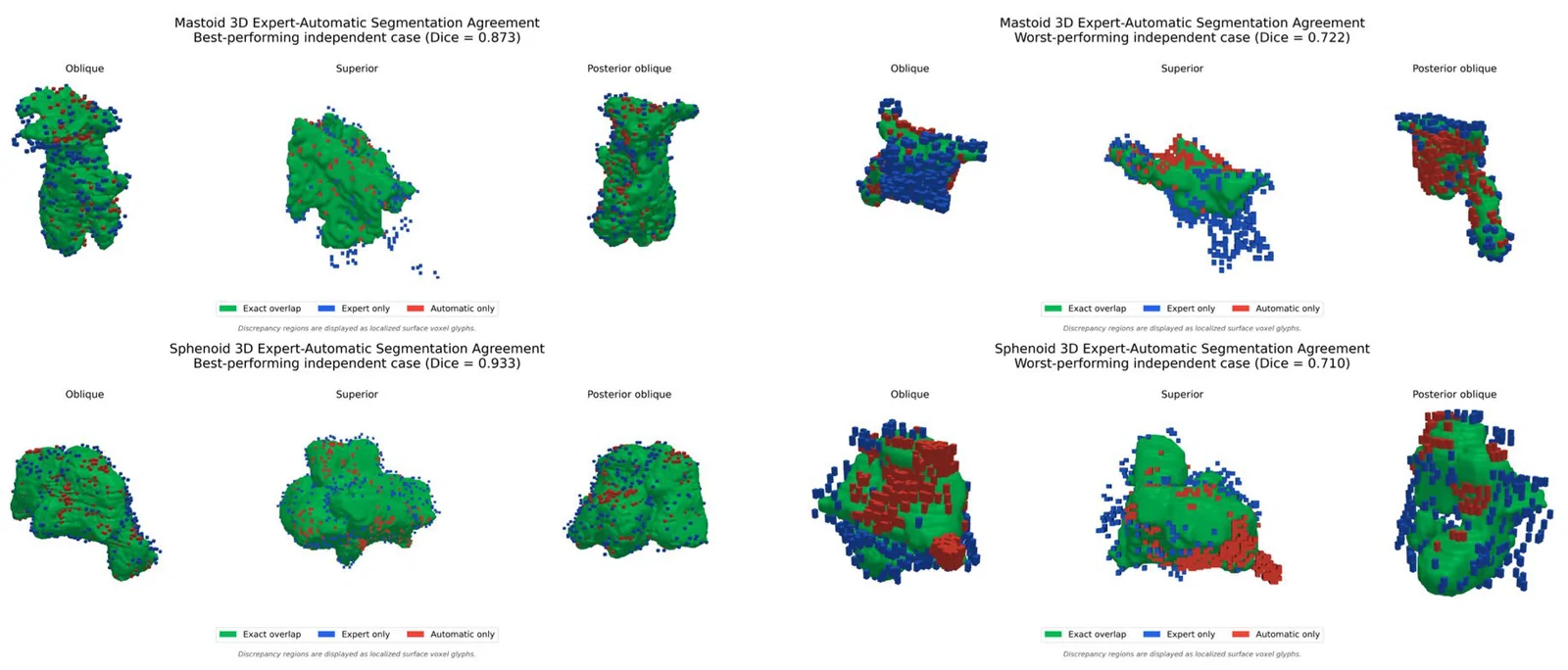
Three-dimensional expert–automatic segmentation agreement in the independent manual evaluation. The best- and worst-performing mastoid cases are shown in the upper panels (Dice = 0.873 and 0.722), and the corresponding sphenoid cases are shown in the lower panels (Dice = 0.933 and 0.710). Each case is displayed in oblique, superior, and posterior-oblique views. Green represents exact voxel overlap between the independent expert mask and automatic prediction, blue represents expert-only regions indicating AI under-segmentation, and red represents automatic-only regions indicating AI over-segmentation. The increased blue and red components in the lower-performing cases illustrate greater boundary disagreement and differences involving peripheral extensions and internal compartment limits. The three-dimensional overlap shown here compares the automatic prediction specifically with the Expert 1 manual mask; therefore, the Dice values displayed above each panel correspond to Expert 1.

The worst-performing sphenoid case demonstrated predominantly conservative AI under-segmentation. The worst-performing mastoid case had a smaller, irregular compartment in which differences in peripheral air cells, bony boundaries, and intracompartmental septa had a proportionally greater effect on Dice. Figure 15 presents 3D renderings of these selected cases using the Expert 1 comparison masks.

### 3.5. Exploratory Volumetric and Densitometric Findings

The final 122 mastoid labels represented anatomical pneumatization compartments rather than air-only masks. The mean and median compartment volumes were 7.13 and 6.62 mL, respectively (range, 0.13–23.33 mL). The mean fractions of voxels categorized as air density, soft-tissue/fluid density, and dense/bony density were 47.8%, 20.2%, and 29.5%, respectively. These findings are consistent with the inclusion of internal bony septa, partial-volume boundaries, and opacified or sclerotic cells according to the anatomical annotation protocol.

Across the 117 sphenoid development labels, the mean and median compartment volumes were 9.40 and 8.77 mL, respectively (range, 1.79–21.82 mL). The mean air-density, soft-tissue/fluid-density, and dense/bony-density fractions were 88.25%, 7.81%, and 3.21%, respectively. Forty-one of one hundred seventeen cases contained at least 95% air-density voxels, and forty of one hundred seventeen contained at least 5% soft-tissue/fluid-density voxels. These fixed HU categories were used only for descriptive exploratory volumetric and densitometric analysis and were not interpreted as diagnostic evidence of inflammation, infection, fungal disease, or neoplasia.

The attenuation findings should be interpreted as compartment-level descriptive characterization rather than tissue classification. HU measurements may vary with CT scanner, tube settings, reconstruction kernel, slice thickness, spatial resampling, and partial-volume effects. The predefined, non-overlapping HU intervals therefore summarize the composition of the expert-defined masks, including air, internal septa, boundary voxels, and opacified content but do not establish a clinical diagnosis.

### 3.6. Segmentation Duration (Ai-Assisted vs. Manual)

Segmentation duration was measured from case loading to successful label submission. Cases affected by technical interruptions or lacking valid timing records were excluded. Because of the small samples and non-normal time distributions, manual and AI-assisted durations were compared using exact two-sided Wilcoxon signed-rank tests.

AI assistance significantly reduced segmentation time for both anatomical targets (Table 3). All matched cases were completed faster using AI-assisted correction. The sphenoid comparison used the same ten cases and the same expert for both workflows, providing a direct paired comparison. The mastoid analysis included seven case-matched observations, but different experts performed the manual and assisted workflows; therefore, this result should be interpreted as exploratory.

Overall, AI assistance reduced the mean segmentation duration by more than 80% for both targets. These findings support its practical value for accelerating clinician-guided segmentation. However, the timing results demonstrate improved workflow efficiency and should not be interpreted as evidence of reduced annotation bias or anchoring bias.

## 4. Discussion

This retrospective pilot study showed that clinician-guided three-dimensional SegResNet workflows could segment mastoid and sphenoid pneumatized compartments on clinical bone CT. The internal-validation Dice was 0.8660 for mastoid and 0.8750 for sphenoid. In independently annotated subsets, mastoid AI-to-expert Dice was 0.8083–0.8097 versus an interobserver Dice of 0.8003; sphenoid AI-to-expert Dice was 0.8339–0.8473 versus an interobserver Dice of 0.9136. Mastoid model disagreement was therefore similar to human disagreement, whereas the sphenoid results indicate room for further model improvement. AI-assisted correction produced higher Dice values (0.9539–0.9691 for mastoid and 0.9347–0.9426 for sphenoid), but these represent workflow agreement rather than independent accuracy because the experts viewed and corrected the prediction, which provided a common starting geometry and could anchor boundary decisions. Independent masks captured differences in thin bony limits, internal septa, peripheral extensions, and opacified or sclerotic cells, explaining their lower overlap. Both models showed predominantly conservative under-segmentation, and improved surface agreement at 2 mm indicated that most discrepancies were localized to boundaries rather than anatomically misplaced. This pattern can be practical in supervised correction because omitted peripheral regions can be added while the clinician retains control of the final mask. The higher assisted mastoid Dice therefore does not demonstrate intrinsic superiority over the sphenoid model.

AI assistance reduced the mean segmentation duration by 81.4% in the paired sphenoid analysis (*p* = 0.0020) and by 86.5% in the exploratory mastoid analysis. The paired sphenoid comparison provides the stronger estimate, whereas the seven-case mastoid analysis involved different experts and should be interpreted cautiously. In both workflows, MONAI Label and 3D Slicer provided an efficient starting mask that remained subject to multiplanar clinician review and correction. The ability to perform training and inference on a local laptop with an 8 GB NVIDIA GPU supports investigator-led feasibility, although anatomical expertise and quality control remain essential.

The sphenoid findings are broadly consistent with recent sinonasal segmentation studies, although differences in modality, target definition, and validation design preclude direct ranking. Darbari Kaul et al. reported a mean Dice of 0.87 for open-source U-Net++ segmentation of the sinonasal cavity on CT [28], close to our internal sphenoid Dice of 0.8750. Gulsen et al. reported a sphenoid Dice of 0.96 using nnU-Net v2 on CBCT after training for 1000 epochs [29]. These methodological and computational differences limit direct comparison with our clinical full-head CT model developed on an 8 GB laptop GPU. Accordingly, these values provide context rather than evidence of equivalence or superiority. Temporal bone deep learning studies have mainly segmented compact structures such as the labyrinth, ossicles, facial nerve, internal auditory canal, and sigmoid sinus. Reported Dice values include 0.75–0.91 for AH-Net [30], 0.6858–0.9329 across Res U-Net, SegResNet, and UNETR [31], and 0.77–0.90 for W-Net [32]. Our independent mastoid Dice of approximately 0.81 lies within this range, but the complete mastoid compartment is more branching, discontinuous, and septated than these compact targets. Comparison with human reproducibility is therefore more informative than comparison with the highest Dice reported for a different structure. Previous mastoid AI work has also focused on classification. Khosravi et al. classified mastoid appearances with approximately 87.7% accuracy [33], but classification does not produce a volumetric compartment mask. Our novelty is not a new architecture because MONAI Label, SegResNet, and clinician-in-the-loop segmentation are established [23,24,26]. It lies in their combined mastoid–sphenoid application with independent manual evaluation, interobserver analysis, timing, and exploratory volumetric characterization.

Skull-base pneumatization is highly variable, and mastoid air cells may extend toward the squamous, zygomatic, petrous, or occipital regions [1,3,8,11]. Although historically uncommon, the term pneumosinus dilatans became more widely recognized from approximately 1989 as CT assessment expanded [5,18,19,34], while its etiology remains unresolved. The present study does not establish a mechanism, but segmentation could support longitudinal assessment of volume, shape, asymmetry, and extension more faithfully than simplified spherical measurements of irregular compartments [4,8,11]. This is relevant because pneumatized spaces may remodel over time under developmental, pressure-related, or local influences, hypotheses that require longitudinal rather than cross-sectional testing. Pneumatization also alters surgical corridors. Temporal-bone extension changes relationships with the tegmen, sigmoid sinus, and petrous apex, while sphenoid hyperpneumatization may affect midline orientation, septal insertion, lateral recesses, wall thickness, and proximity to the internal carotid arteries or optic nerves [13,18,19]. Three-dimensional masks may support preoperative review and longitudinal morphometry, but the present HU analysis remains descriptive and cannot diagnose inflammation, fungal disease, or tumor.

With external validation, these masks could support patient-specific visualization, neuronavigation, educational models, and quantitative analysis of septa, wall morphology, or longitudinal changes [35,36,37,38]. Such clinician-centered imaging could also provide a foundation for future radiomic decision-support research [36]. Augmented reality and robotic integration remain future possibilities rather than outcomes demonstrated in the present study [35,37,38,39,40].

Several limitations must be acknowledged. This was a retrospective, single-center pilot study without external testing. Although the reserved cohorts were separated at the patient level and excluded from training, the independent subsets were small (eight mastoid and ten sphenoid cases). The larger assisted evaluations used corrected AI predictions and are susceptible to anchoring; they measure correction–workflow agreement rather than independent accuracy. The mastoid workflow uses rule-based side cropping rather than end-to-end full-head localization. The inclusion of septa and opacified or sclerotic cells meant that the target represented an anatomical compartment rather than only air-density voxels and made some boundaries observer-dependent. Scanner heterogeneity may also have affected performance, and HU analysis was not validated diagnostically. Finally, sphenoid timing was paired, whereas mastoid timing involved different experts and fewer matched cases and therefore remains exploratory.

Future work should prioritize larger independently annotated cohorts, external multicenter validation, and prospective same-expert timing studies across different scanners and clinical presentations. Automated mastoid localization could enable end-to-end inference from full-head CT, while longitudinal studies should assess whether the workflow can reliably quantify changes in pneumatization volume and extension over time. As part of the first author’s ongoing doctoral research, this workflow will be extended to multicenter studies of skull base tumors, with particular emphasis on longitudinal changes in pneumatization, tumor morphology, and the surrounding peritumoral imaging environment.

## 5. Conclusions

This study demonstrates that a clinician-guided, clinician-centered workflow can support deep learning segmentation and exploratory volumetric assessment of skull base pneumatized compartments on bone CT datasets. The mastoid model achieved an internal-validation Dice of 0.8660. In the AI-assisted evaluation, the mean Dice was 0.9691, with a Jaccard index of 0.9419, median HD95 of 0.9468 mm, and mean surface distance of 0.3099 mm. The sphenoid model achieved an internal-validation Dice of 0.8750 and an AI-assisted mean Dice of 0.9426, with a Jaccard index of 0.8937, median HD95 of 1.875 mm, and median average symmetric surface distance of 0.331 mm. These assisted results were analyzed separately from the independent manual evaluation because the reference masks were obtained by correcting displayed AI predictions.

In the independent manual subsets, AI-to-expert Dice was 0.8083–0.8097 for the mastoid and 0.8339–0.8473 for the sphenoid. At a 2 mm tolerance, AI-to-expert surface Dice reached 0.903–0.913 and 0.894–0.903, respectively, indicating that most discrepancies were localized near anatomical boundaries. Both models demonstrated predominantly conservative under-segmentation, particularly around peripheral extensions and internal bony septa. AI assistance reduced mean segmentation duration by 81.4% in the paired sphenoid analysis (*p* = 0.0020) and by 86.5% in the exploratory mastoid analysis (*p* = 0.0156).

Clinician-guided review of imaging data and model outputs may reinforce the physician’s understanding of patient-specific anatomical boundaries, thereby supporting continuous medical education and preoperative preparation. Deep-learning-assisted segmentation may improve the visualization and quantitative assessment of individual skull base pneumatization patterns and contribute to a more personalized approach to surgical planning. With appropriate validation, these tools may support risk stratification and the delineation of anatomically complex surgical boundaries. However, increasing algorithmic complexity should not displace clinical judgment: physicians must remain central to the design, supervision, interpretation, and responsible application of these systems.

## Figures and Tables

**Table 1:** The exploratory distribution of pneumatization extensions among the 122 mastoid side-cases, including extensions beyond the central mastoid compartment.

Exploratory Extension Proxy	Count Among 122 Mastoid Side-Cases (n)
Superior extension toward temporal squama	5
Anterior/zygomatic extension	8
Posterior extension	16
Medial extension toward petrous bone	29

**Table 2:** Best- and worst-performing independent segmentations for the mastoid and sphenoid targets.

Target	Performance	Case	AI vs. Expert 1	AI vs. Expert 2	Mean Dice
**Mastoid**	Best	M02	0.873	0.874	**0.874**
**Mastoid**	Worst	M03	0.722	0.743	**0.733**
**Sphenoid**	Best	SP10	0.933	0.920	**0.927**
**Sphenoid**	Worst	SP02	0.710	0.712	**0.711**

**Table 3:** Manual versus AI-assisted segmentation times.

Target	Comparison	n	Mean Time	Median Time	Range	Mean Reduction	*p*-Value
**Mastoid**	Manual	7	8.25 min	7.00 min	1.49–20.54 min	—	—
**Mastoid**	AI-assisted	7	1.11 min	1.06 min	0.51–1.68 min	**86.5%**	**0.0156**
**Sphenoid**	Manual	10	7.87 min	6.15 min	3.44–24.91 min	—	—
**Sphenoid**	AI-assisted	10	1.46 min	1.27 min	1.04–2.21 min	**81.4%**	**0.0020**

## Data Availability

The data used in this study are not publicly available because of privacy and ethical restrictions but may be made available by the corresponding author upon reasonable request and with permission from the data provider. A sanitized, code-only version of the study workflow is publicly available on GitHub.

## Supplementary Materials

The following supporting information can be downloaded at: https://www.mdpi.com/article/10.3390/life16081284/s1. MONAI Label source code: https://project-monai.github.io/ (accessed on 31 July 2026), https://github.com/Project-MONAI/MONAILabel (accessed on 31 July 2026), Sanitized source code for the study workflow, with patient data excluded: https://github.com/ionescucristian1/skullbase-pneumatization-ai-code_SANITIZED_CODE_ONLY_20260628.git (accessed on 31 July 2026), https://doi.org/10.5281/zenodo.21066337 (accessed on 31 July 2026).

## Copyright Statement

All figures, tables, schemes, workflow diagrams, segmentation renderings, and image-derived materials included in this manuscript are original materials created by the authors from anonymized study data or from the authors’ own workflow documentation. No previously published copyrighted figures, tables, schemes, or substantial text excerpts have been reproduced. Any external concepts, methods, software tools, or scientific findings discussed in the manuscript have been appropriately cited in the reference list. The authors confirm that they hold the necessary rights to submit the original materials included in this article for publication.

## List of Abbreviations

AI	artificial intelligence
ASSD	average symmetric surface distance
CBCT	cone-beam computed tomography
CNN	convolutional neural network
CT	computed tomography
DICOM	Digital Imaging and Communications in Medicine
Dice	Dice similarity coefficient
DiceCE	Dice and cross-entropy loss
ENT	ear, nose, and throat/otorhinolaryngology
FN	false negative
FP	false positive
GPU	graphics processing unit
HD95	95th percentile Hausdorff distance
HRCT	high-resolution computed tomography
HU	Hounsfield unit
IoU	intersection over union
MRI	magnetic resonance imaging
MONAI	Medical Open Network for Artificial Intelligence
NIfTI	Neuroimaging Informatics Technology Initiative
nnU-Net	no-new-Net/self-configuring U-Net framework
PPV	positive predictive value
RVE	relative volume error
SCJU	County Emergency Clinical Hospital
SegResNet	segmentation residual network
TP	true positive
U-Net	U-shaped convolutional neural network architecture
3D	three-dimensional
2D	two-dimensional
mL	milliliter
mm	millimeter
kVp	kilovoltage peak
