# Supplementary material for: Clinician-Guided Deep Learning Segmentation of Skull Base Pneumatization on Computed Tomography Using 3D Slicer and MONAI Label
Source: Life (Basel). 2026 Aug 3;16(8):1284. doi: 10.3390/life16081284 (PMC13514538; doi:10.3390/life16081284)
Supplement: Supplementary file 1 [file life-16-01284-s001.zip › life-4439839-supplementary.pdf]

Supplementary Materials

MONAI Label source code:
https://project-monai.github.io/
(Accessed on 2 August 2026)
https://github.com/Project-MONAI/MONAILabel
(Accessed on 2 August 2026)
Sanitized source code for the study workflow, with patient data excluded:
https://github.com/ionescucristian1/skullbase-pneumatization-ai-code_SANITIZED_CODE_ONLY_20260628.git (Accessed on 2 August 2026)
https://doi.org/10.5281/zenodo.21066337

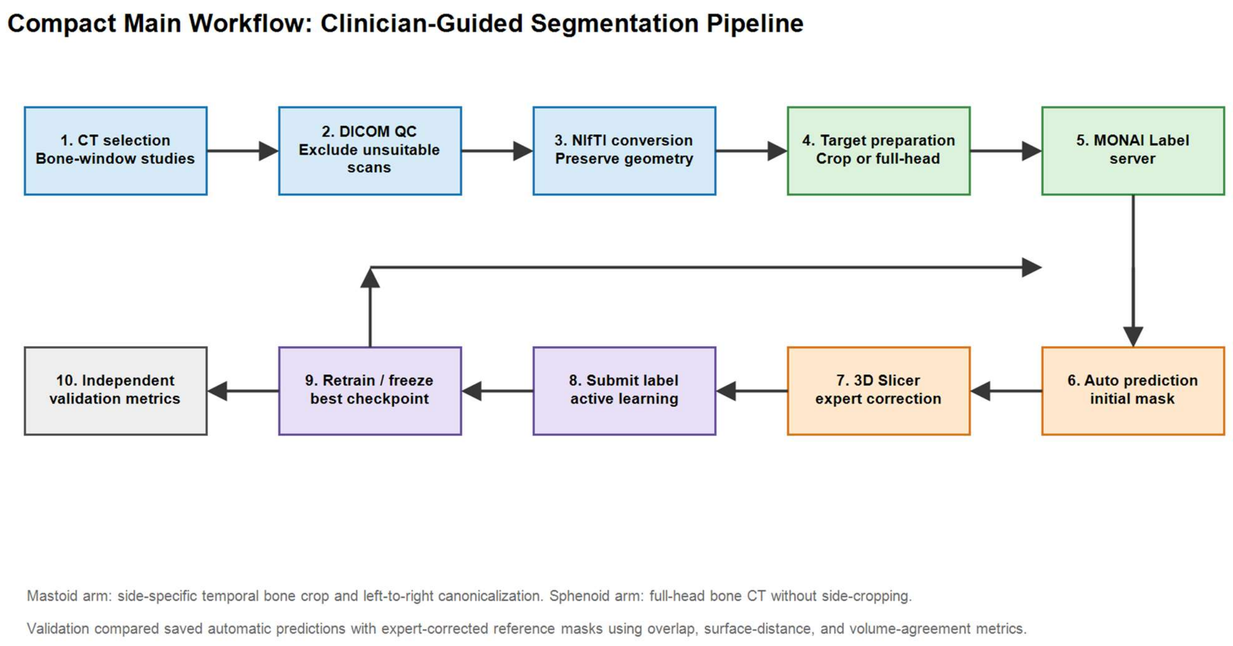

Figure S1. Clinician-guided segmentation pipeline.

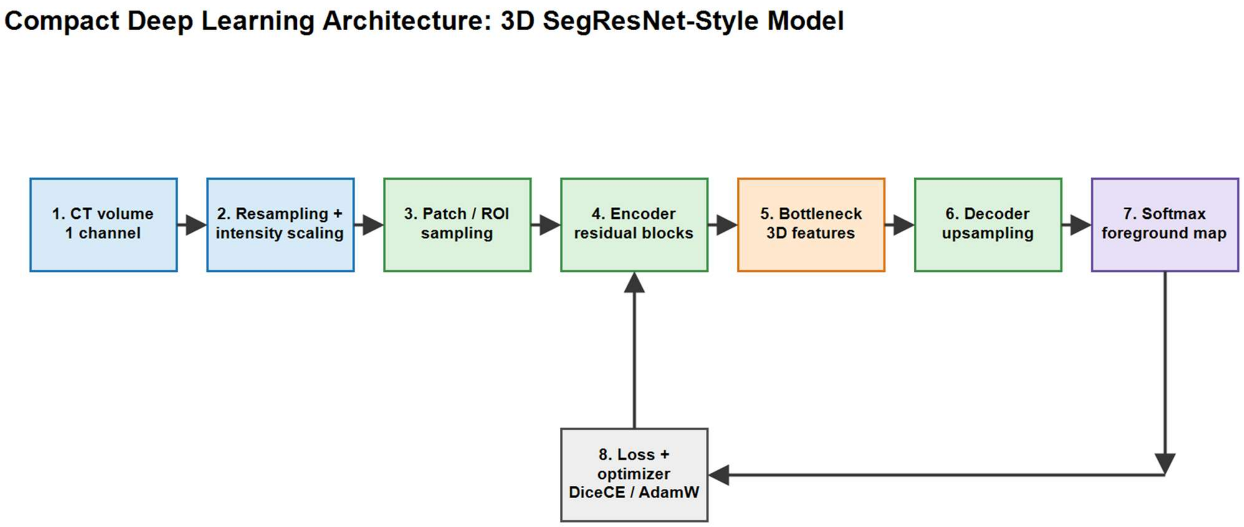

Figure S2. Simplified three-dimensional SegResNet architecture pipeline.

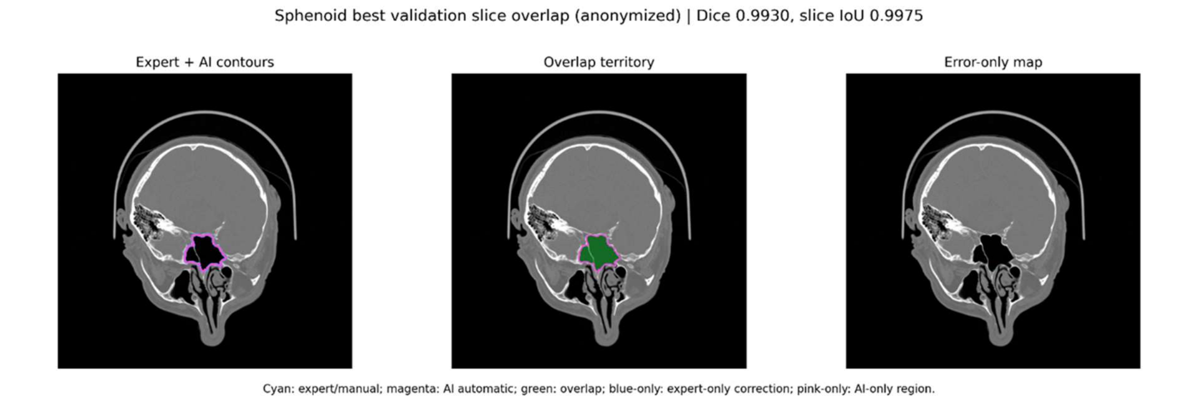

Figure S3. Best Sphenoid automatic vs. manual overlap on native bone CT scans.

Figure S3. Best sphenoid automatic versus manual overlap on native bone CT scans. Cyan outlines the expert/manual mask, magenta outlines the automatic mask, green indicates overlap, blue indicates expert-only regions, and pink indicates automatic-only regions.

CT Slice Burden and Segmented-Slice Extent

The two study arms had similar median z-axis image depths but differed in anatomical preparation and segmented target extent. After exclusion filtering, mastoid side-crop CT volumes had a median of 281.5 axial slices per side-case, whereas sphenoid full-head CT volumes had a median of 260 axial slices per CT examination. The original in-plane matrix of the sphenoid CT examinations was 512 × 512, whereas mastoid side-cases were cropped around one temporal bone to reduce non-target anatomy.

The final mastoid label set contained 6630 segmented axial slices across 122 side-cases, with a median of 56.5 segmented slices per side-case. The current sphenoid label set contained 3977 segmented axial slices across 117 expert-submitted full-head CT labels, with a median of 36 segmented slices per CT examination. These values illustrate that both targets were volumetric structures rather than isolated single-slice masks, while the mastoid segmentation target generally occupied a longer slice extent than the sphenoid sinus (Table S1).

Table S1. CT slice burden and segmented-slice extent for the mastoid and sphenoid study arms. Abbreviation: mm, millimeters.

Slice-burden metric	Mastoid arm	Sphenoid arm
**Analyzed image unit**	Side-specific temporal bone crop	Full-head bone CT
**Active/final image count for slice statistics**	122 locked side-cases after exclusions	117 full-head CT volumes with submitted labels
**Median CT slices per image volume**	281.5 slices	260 slices
**CT slice range**	137–375 slices	66–375 slices
**Submitted labels used for segmented-slice count**	122 final mastoid labels	117 final sphenoid labels
**Total segmented axial slices**	6630	3977
**Median segmented slices per case**	56.5	36.0
**Mean segmented slices per case**	54.3	34.0
**Segmented-slice range**	13–89	8–64
**Median segmented z-span**	38.1 mm	25.5 mm
**Mean segmented z-span**	36.3 mm	26.5 mm

Principal Mastoid Training Runs

Table S2. Mastoid training epochs, including original development, integrity investigation, and recovery runs.

Development stage	Epochs planned/reached	Recorded duration	Best internal-validation Dice	Role
Early development	40/40	49 min 41 s	0.8613 at epoch 35	Initial useful model
Parameter refinement	40/40	2 h 50 min 25 s	0.8840 at epoch 20	Early improvement
Strong single-side model	40/40	4 h 15 min 24 s	0.9244 at epoch 40	Right-side assisted-mask basis

Preserved development checkpoint	40/30	3 h 02 min 58 s	0.9291 at epoch 5	Incomplete run; checkpoint preserved
Strong completed model	40/40	3 h 52 min 27 s	0.9285 at epoch 35	Completed historical high-performing model
Later development	50/50	5 h 25 min 21 s	0.8829 at epoch 15	Validation decline
Data-integrity investigation	15/15	1 h 35 min 57 s	0.4256 at epoch 15	Prompted label and orientation review
Pre-recovery run	25/25	2 h 20 min 49 s	0.0719 at epoch 10	Excluded from final model claims
Left-side recovery baseline	40/40	3 h 38 min 13 s	0.4885 at epoch 25	Post-audit recovery
Right-side recovery check	15/15	49 min 37 s	0.6448 at epoch 10	Confirmed usable right-side pathway
Left-side refinement	50/50	2 h 05 min 31 s	0.7644 at epoch 45	Corrected-label refinement
Final left-side recovery	60/60	3 h 31 min 46 s	0.8437 at epoch 35	Recovered left-side model
Combined left–right training	60/60	5 h 59 min 26 s	0.8644 at epoch 55	Integration of curated sides
Final combined refinement	40/40	3 h 53 min 35 s	0.8660 at epoch 30	Frozen model used for evaluation

Detailed Sphenoid Training Log

The sphenoid model was developed independently from the mastoid model and did not use mastoid pretrained weights. Sequential refinements were initialized only from previous sphenoid checkpoints. The complete sphenoid development history comprised 332 recorded epochs across exploratory, intermediate, and final refinement runs. This historical total should not be interpreted as a single uninterrupted 332-epoch training session or as equal exposure of every case to 332 epochs.
The final development dataset contained 117 expert-submitted sphenoid labels, including 102 training and 15 internal-validation examinations. The model used a binary three-dimensional SegResNet configuration with background and sphenoid output channels. Full-head CT volumes were resampled to $0.5 \times 0.5 \times 0.625$ mm. Training used $64 \times 64 \times 64$-voxel regions, four sampled regions per image, a 3:1 positive-to-negative sampling ratio, a batch size of 1, and a final learning rate of $5 \times 10{-}5$. Memory-safe full-volume validation was performed using sliding-window inference.

Longer training did not consistently improve generalization. In the completed 70-label run, internal-validation Dice peaked at 0.6372 at epoch 45 but decreased to 0.3606 by epoch 60, despite a final training Dice of 0.8963. Similarly, in the selected 117-label refinement, validation Dice reached 0.8750 at epoch 10 and decreased to 0.8308 by epoch 15. The epoch-10 checkpoint was therefore preserved and frozen rather than continuing automatically to the planned maximum.
The directly traceable sphenoid warm-start lineage comprised 215 selected epochs across sequential dataset-expansion and refinement stages. This differs from the complete historical total of 332 recorded epochs, which also includes exploratory, interrupted, superseded, and non-lineage epochs. Internal-validation Dice was used for checkpoint selection, whereas the reserved AI-assisted and blinded-manual evaluations were performed after model freezing and were not used to modify model weights or training parameters.

Recorded durations varied according to dataset size, validation workload, and configuration. They describe performance on the local computational environment and should not be interpreted as platform-independent benchmarks.

Table S3. Sphenoid training durations and epochs.

Development stage	Epochs planned/reached	Recorded duration	Best internal-validation Dice	Role
17-label pilot	25/25	1 h 02 min 16 s	0.8062	Initial developmental run
60-label development	50/50	6 h 24 min 20 s	0.4716 at epoch 50	Dataset-expansion experiment
70-label development	60/60	9 h 00 min 01 s	0.6372 at epoch 45	Demonstrated late validation decline
107-label development	35/35	8 h 34 min 32 s	0.6494 at epoch 15	Intermediate refinement
117-label selected refinement	35/15	3 h 49 min 45 s to epoch 15	**0.8750 at epoch 10**	Frozen model used for evaluation

Table S4. Image and submitted-label characteristics for the sphenoid arm. Abbreviation: mm, millimeters.

Parameter	Value
**CT matrix size**	512 × 512 × Z in all analyzed cases
**Mean CT slice count**	240.6
**Median CT slice count**	260
**CT slice count range**	66–375
**Mean segmented sphenoid slices**	34.0
**Median segmented sphenoid slices**	36
**Segmented sphenoid slice range**	8–64
**Mean segmented-slice fraction**	14.18% of CT slices
**Median segmented-slice fraction**	13.85% of CT slices
**Median z-spacing**	0.625 mm
**Median in-plane spacing**	approximately 0.488 × 0.488 mm

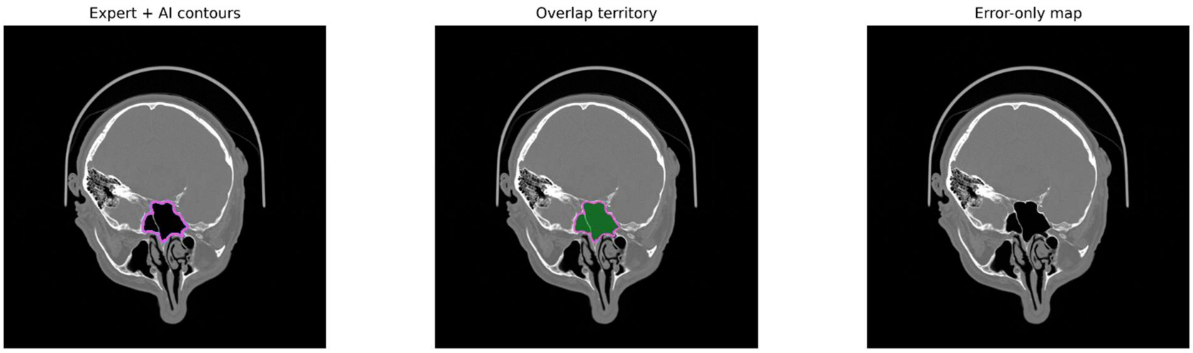

Figure S3. Best Sphenoid automatic vs. manual overlap on native bone CT scans.

Technical Apparatus and Software Environment

**Component**	**Specification**
CT imaging systems	Siemens Healthineers SOMATOM X.cite; GE Healthcare Revolution Frontier; GE Healthcare Optima CT660
Source imaging format	Digital Imaging and Communications in Medicine (DICOM)
Segmentation and training format	Neuroimaging Informatics Technology Initiative (NIfTI)
Operating system	Microsoft Windows 11 Home
Processor	AMD Ryzen 9 8940HX with Radeon Graphics
System memory	32 GB RAM
Deep-learning GPU	NVIDIA GeForce RTX 5070 Laptop GPU
GPU memory	8 GB VRAM (8151 MiB reported)
Secondary graphics adapter	AMD Radeon Graphics, used for display support only
GPU computation	NVIDIA CUDA-enabled acceleration
CUDA runtime	CUDA 12.8
Python	Python 3.10.20
Environment management	Anaconda installation; dedicated monailabel-py310 environment created using conda 25.11.1
Deep-learning framework	PyTorch 2.11.0+cu128
Medical-imaging framework	MONAI 1.5.2
Interactive AI platform	MONAI Label 0.8.5
Segmentation software	3D Slicer 5.10.0
Network architecture	Three-dimensional SegResNet
Training configuration	Single NVIDIA GPU; training and validation batch size of one
Image-processing libraries	SimpleITK 2.5.3; NiBabel 5.4.2; pydicom 2.4.4
Numerical and statistical libraries	NumPy 1.26.4; SciPy 1.15.3; pandas 2.3.3
Analytical libraries	scikit-image 0.25.2; scikit-learn 1.7.2

Command-line environment	Windows PowerShell 5.1
Initial text/configuration editing	Notepad++ 8.9.3
Code availability	Sanitized, code-only repository hosted on GitHub
Data protection	No DICOM images, NIfTI volumes, expert labels, patient identifiers, training checkpoints, or identifiable file names were included in the public repository
AI-assisted development tools	OpenAI Codex and Anthropic Claude were used for coding support, workflow documentation, and the organization of verified outputs
Human oversight	AI tools did not select patients, establish anatomical boundaries, create independent expert reference masks, determine exclusions, or make clinical interpretations
Platform consistency	Mastoid and sphenoid models were developed, trained, and evaluated on the same local hardware and software platform

Note: Software versions were verified from the dedicated project environment and local installation records. The MONAI environment was created before the recorded training runs. Training logs confirmed CUDA execution on the NVIDIA GeForce RTX 5070 Laptop GPU. All anatomical annotations, inclusion and exclusion decisions, model acceptance decisions, and scientific interpretations remained under clinician supervision.

List of Abbreviations

AI: artificial intelligence
ASSD: average symmetric surface distance
CBCT: cone-beam computed tomography
CNN: convolutional neural network
CT: computed tomography
DICOM: Digital Imaging and Communications in Medicine
Dice: Dice similarity coefficient
DiceCE: Dice and cross-entropy loss
ENT: ear, nose, and throat / otorhinolaryngology
FN: false negative
FP: false positive
GPU: graphics processing unit
HD95: 95th percentile Hausdorff distance
HRCT: high-resolution computed tomography
HU: Hounsfield unit
IoU: intersection over union
MRI: magnetic resonance imaging
MONAI: Medical Open Network for Artificial Intelligence
NIfTI: Neuroimaging Informatics Technology Initiative
nnU-Net: no-new-Net / self-configuring U-Net framework
PPV: positive predictive value
RVE: relative volume error
SCJU: County Emergency Clinical Hospital
SegResNet: segmentation residual network
TP: true positive
U-Net: U-shaped convolutional neural network architecture
3D: three-dimensional
2D: two-dimensional

mL: milliliter
mm: millimeter
kVp: kilovoltage peak
